# Diversity of *Sordariales* Fungi: Identification of Seven New Species of *Naviculisporaceae* Through Morphological Analyses and Genome Sequencing

**DOI:** 10.3390/jof11120880

**Published:** 2025-12-12

**Authors:** Narumon Tangthirasunun, Valérie Gautier, Christophe Lalanne, Lucas Bonometti, Sandrine Cros-Arteil, Richard D. Hayes, Sarah Calhoun, Robert Riley, Jasmyn Pangilinan, Anna Lipzen, Vivian Ng, Igor V. Grigoriev, Pierre Gladieux, Tatiana Giraud, Philippe Silar

**Affiliations:** 1 Laboratoire Interdisciplinaire des Energies de Demain, Université Paris Cité, UMR CNRS 8236, 75205 Paris Cité CEDEX 13, France; 2 Department of Biology, School of Science, King Mongkut’s Institute of Technology Ladkrabang, Bangkok 10520, Thailand; 3 Plant Health Institute Montpellier, University of Montpellier, INRAE, CIRAD, IRD, Institut Agro, 34398 Montpellier, France; 4 U.S. Department of Energy Joint Genome Institute, Lawrence Berkeley National Laboratory, Berkeley, CA 94720, USA; 5 Department of Plant and Microbial Biology, University of California Berkeley, Berkeley, CA 94720, USA; 6 Ecologie Société et Evolution, CNRS, Université Paris-Saclay, AgroParisTech, 91198 Gif-sur-Yvette, France

**Keywords:** *Sordariales*, *Naviculisporaceae*, phylogenomics, new species

## Abstract

Thanks to next-generation sequencing (NGS) technologies, the diversity of fungi can now be investigated through the analysis of their genome sequences. *Naviculisporaceae* is a family within the *Sordariales*, whose diversity is not well-known, with only one genome sequence published for this family. Here, we report on the isolation and cultivation of 20 new strains of *Naviculisporaceae*. Their genome sequences, as well as those of the five commercially available strains, were determined, thus providing complete genome sequences for 25 new *Naviculisporaceae* strains. Species delimitation was conducted using a combination of (1) ITS + LSU phylogenetic analysis of the new isolates along with other known species of the family, (2) comparisons between DNA barcode sequences of the new strains with those of the known species, and (3) average genome-wide nucleotide identity calculation. We built a phylogenomic tree and studied the organization of the mating-type locus. In vitro fruiting was obtained for 16 strains, enabling the definition of seven new species, namely *Pseudorhypophila gallica*, *Pseudorhypophila guyanensis Rhypophila alpibus*, *Rhypophila brasiliensis*, *Rhypophila camarguensis*, *Rhypophila reunionensis* and *Rhypophila thailandica*, as well as two new combinations, namely *Pseudorhypophila latipes* and *Pseudorhypophila oryzae*. Eight strains for which in vitro fruiting was not obtained may belong to additional new species. These results expand the known diversity of the *Naviculisporaceae* and greatly enlarge the genomic data available for the family.

## 1. Introduction

Complete genome sequences, when combined with crossing assays and morphological analyses, enable accurate species delimitation. In particular, the average nucleotide identity (ANI) can be very useful to delimit species using genome-wide sequences [1,2,3]. In most cases, a nucleotide divergence threshold of 1% allows clear species delimitation using the FungANI programme: strains with ANI values above 99.5% consistently belong to the same species, whereas those with ANI values below 99% usually correspond to distinct species [2]. Genome analyses often reveal unexpected or cryptic diversity in fungi, uncovering distinct genetic lineages that remain indistinguishable based on morphology alone (see [4,5,6,7,8,9,10] for examples in the *Sordariales*). Beyond taxonomy, access to full genomes also provides insights into the mating-type locus structure, contributing to our understanding of the breeding system (e.g., homothallism vs heterothallism). This, for example, can help to explain fertility or sterility observed under culture conditions. Moreover, genome availability opens avenues for mining enzymes of interest, as well as regulatory and secondary metabolite pathways.

The *Naviculisporaceae* is a family within the *Sordariales* (*Sordariomycetes*, *Ascomycota*) that has recently been defined through molecular phylogenies using four DNA barcodes: the internal transcribed spacer region (ITS), and parts of the nuclear rDNA large subunit (LSU), the RNA polymerase II subunit 2 (*RPB2*) and the β-tubulin (*TUB2*) genes [11]. It currently contains four recognized genera, *Areotheca*, *Naviculispora*, *Pseudorhypophila* and *Rhypophila*, which collectively contain 11 recognized species: two species of *Areotheca*, one of *Naviculispora*, four of *Pseudorhypophila* and four of *Rhypophila*. *Naviculisporaceae* also includes three species of *Arnium* and one species of *Gilmaniella*, of which all need taxonomic revision [11,12,13]. It is likely that additional species will be added to this family as more strains are analyzed and more genomes are sequenced. To date, only one *Naviculisporaceae* strain has been sequenced and published: PSN293. It was isolated in 2017 from donkey dung collected in the Auvergne region of central France [14]. Its genome was sequenced under the name *Podospora decipiens* (G. Winter ex Fuckel) Niessl [14], which is the old name for this species, now known as *Rhypophila decipiens* (G. Winter) Y. Marín, A.N. Mill. And Guarro [11].

*Sordariales* fungi occur in a wide range of biotopes and rank among the most wide-spread fungi present in soils [15]. They are also commonly found in the dung of herbivores [16]. Yet, their actual phylogenetic and morphological diversity remains largely unexplored [17]. In particular, genome sequences are unavailable for many species. To address this gap, we undertook a comprehensive sampling effort to characterize the diversity of *Sordariales* in both soil and dung environments. For soil, we developed a dedicated method to facilitate their recovery [18], whereas traditional moist chamber techniques were used to isolate species from dung. Using these approaches, we obtained the *Naviculisporaceae* species that appeared to be new to science. Here, we report the genome sequences of 25 additional *Naviculisporaceae* strains, including five commercially available ones and twenty newly isolated ones. Morphological descriptions of the sexual reproductive structures are provided for the 16 strains that proved fertile in laboratory conditions. These analyses enabled us to identify seven new *Naviculisporaceae* species and to establish new combinations for *Zopfiella latipes* and *Apodus oryzae*, as *Pseudorhypophila latipes* comb. nov. and *Pseudorhypophila oryzae* comb. nov., respectively. Altogether, these findings significantly expand the known diversity of the family.

## 2. Materials and Methods

### 2.1. Sample Collection, Fungal Isolation and Availability

Strains CBS 256.59 and CBS 365.69 were purchased from the Westerdjik Institute (https://wi.knaw.nl/, accessed on 1 June 2021). Strains IMI229743, IMI229747 and IMI350600 were purchased from CABI (https://www.cabi.org/, accessed on 1 June 2021). All the other strains were newly isolated for this study. Soil and dung samples were collected and stored at 4 °C, until they were processed according to previously described protocols [18,19], to recover potential *Sordariales* species. Newly obtained strains were isolated by germinating complete asci or batches of several ascospores to recover *mat1-1*/*mat1-2* heterokaryons for heterothallic species. DNAs were extracted using the rapid method previously described in ref. [18] and were subjected to internal transcribed spacer (ITS) sequencing. Taxonomic assignment to the *Naviculisporaceae* was performed by BLAST analysis of the ITS sequences at the National Centre for Biotechnology Information (NCBI) querying the ITS Fungi RefSeq database (https://www.ncbi.nlm.nih.gov/bioproject/PRJNA177353/, accessed on 1 June 2025). Table 1 provides the origin and main characteristics of the strains used in this study.

The holotype specimens for the new species were deposited in the herbarium of the “Museum National d’Histoire Naturelle” (MNHN, Paris, France). All the newly isolated strains, including the ex-holotype living cultures for the new species, were deposited in the “Centre International de Ressources Microbiennes—Champignons Filamenteux” (CIRM-CF, INRAE, Marseille France).

### 2.2. DNA Isolation for Genome Sequencing, Next-Generation Sequencing and Genome Assembly

DNA for genome sequencing by Novogene (Cambridge, UK) and the Biomics facility (Institut Pasteur, Paris, France) was extracted using the NucleoSpin^®^ Soil from Macherey Nagel (Düren, Germany) and libraries were prepared by the sequencing facilities. For genome sequencing by the JGI, DNA was extracted and libraries were prepared following “Method 4” described in the Supplementary Materials of Refs. [14,20].

For genome sequenced by Novogene (Cambridge, UK) and Biomics (project B16526), DNA was submitted to 2 × 150 bp Novaseq Illumina sequencing. Sequence reads were assembled with Unicycler (Galaxy Version 0.5.0+galaxy1) using the default parameters [21]. Although designed to assemble prokaryotic genomes, this programme works well with fungal genomes, as demonstrated by the BUSCO score (Table S1; [2]).

For the genomes sequenced by JGI, Illumina 400 bp Regular Fragment libraries were sequenced using the NovaSeq S4 in 2 × 150 bp paired reads. All raw Illumina sequence data were filtered for artefacts/process contamination using BBTools version 38.79 kmercountexact.sh (http://sourceforge.net/projects/bbmap, accessed on 11 October 2025). Two million reads were subsampled to assemble mitochondria from the filtered reads using the GetOrganelle toolkit [22], and any organelle-matching reads were removed. The remaining reads were then k-mer-matched against the resulting contigs using BBTools version 38.79 with bbduk.sh [23]. An assembly of the target genome was generated using a 20 million read-pair subsample of the resulting non-organelle reads with SPAdes v3.15.2 [24].

For transcriptomes, stranded RNASeq libraries were sequenced using Illumina NovaSeq S4, filtered for artefacts and contaminants using BBDuk, trimmed for quality using the phred Q6, and assembled using Trinity v.2.11.0 [25]. Genomes were annotated using the JGI Annotation pipeline [26].

Raw data and genome assemblies are available under the GenBank Bioproject accession numbers listed in Table S1.

### 2.3. Phylogenetic Analyses

Table 2 provides the ITS and rDNA large subunit (LSU) GenBank accession numbers for the previously known sequences used in the combined ITS + LSU phylogenetic analysis. ITS + LSU sequences of the newly sequenced strains and that of PSN293 were manually extracted from the genome assemblies. *Apodospora peruviana* CBS 118,394 was chosen as the outgroup for this analysis, as it was shown to be the closest relative of PSN293 [14]. The sequences were aligned using MAFFT V7 with the default parameters [27], and maximum likelihood phylogeny was computed by IQ-tree using the default parameters and 1000 ultrafast bootstraps [28].

### 2.4. Phylogenomic Analyses

We used sequences of BUSCO v.5.5.0 genes to infer phylogenetic relationships. We identified 2798 single-copy orthologs among BUSCO genes (Benchmarking Universal Single-Copy Orthologs). Sequences for each orthogroup were aligned at the codon level (i.e., keeping sequences in coding reading frame) with TranslatorX 1.1 [30], using MAFFT v7 [27] as the aligner and default parameters for Gblocks 0.91b [31]. Sequences were concatenated, and a maximum likelihood phylogeny was inferred using raxml-ng v.1.2.0 [32] with model GTR + G, ten parsimony starting trees, and 100 bootstrap replicates.

The ANI were calculated using FungANI with the default parameters [2].

### 2.5. Mating-Type Locus Annotation

The mating-type loci were manually annotated to ensure correct annotations.

### 2.6. Morphological Analyses

Five media (M2 minimal medium, Oat Meal Agar, V8 Agar, M0 + miscanthus and M0 + hay) and two temperatures (room temperature and 27 °C) were tested for ascocarp production. Measurements of perithecium diameters were made on 10 randomly selected fruiting bodies and ascospore measurements were made on 50 randomly selected spores originating from different ascocarps.

Compositions of the M2 and M0 media have been previously published [19]. M0 + miscanthus and M0 + hay media were obtained by overlaying the M0 plates with 0.05 g of sterilized shredded miscanthus or hay, respectively. V8 medium is 200 mL/L of V8 juice, 3 g/L of CaCo_3_ and 15 g/L of agar. Oat Meal Agar is 20 g/L of oatmeal and 15 g/L of agar.

## 3. Results

### 3.1. Fungal Isolates and Species Delimitation

Table 1 summarizes the dates of isolation, geographical origins, and substrates of the strains purchased or isolated for this study. Table 1 also includes the main features of their genome assemblies (detailed statistics of the genome sequences used for phylogenomic analyses are presented in Table S1). As shown in Table S1, genome assemblies were generally of high quality, with BUSCO scores exceeding 97% in all cases, except for the previously published *Diplogelasinospora grovesii* CBS 340.73 genome, which was used as an outgroup in the phylogenomic analyses.

From most *Naviculisporaceae* genomes, we were able to retrieve the ITS + LSU region directly from the assemblies. When this region was absent, reads mapping to rRNA loci were extracted with Bowtie2 version 2.5.0 [33] and reassembled with Unicycler [21]. These sequences, combined with ITS + LSU data already available for *Naviculisporaceae* (Table 2), were used in a first step for species delimitation and assignment to known species. *Apodospora peruviana* CBS 118,394 was used as an outgroup for this analysis, as this strain had previously been shown to be the closest relative of the sequenced PSN293 [14]. For this strain, the ITS and LSU sequences obtained from the genome assembly were identical to those deposited in GenBank (GenBank accession numbers in Table 2). Finer species delimitation and assignment were then made by comparing with BLAST some genes of the investigated strains to orthologs in their closest relatives whose sequences are present in GenBank. These included the glyceraldehyde 3-phosphate dehydrogenase (*gpd*) gene, the RNA polymerase II subunit (*RPB2*) gene, the beta-tubulin (*TUB2*) gene, and the sordarin secondary metabolite gene cluster. Finally, for fine comparison between genome sequences of closely related strains, ANI was calculated with FungANI [2].

**Figure 1:**
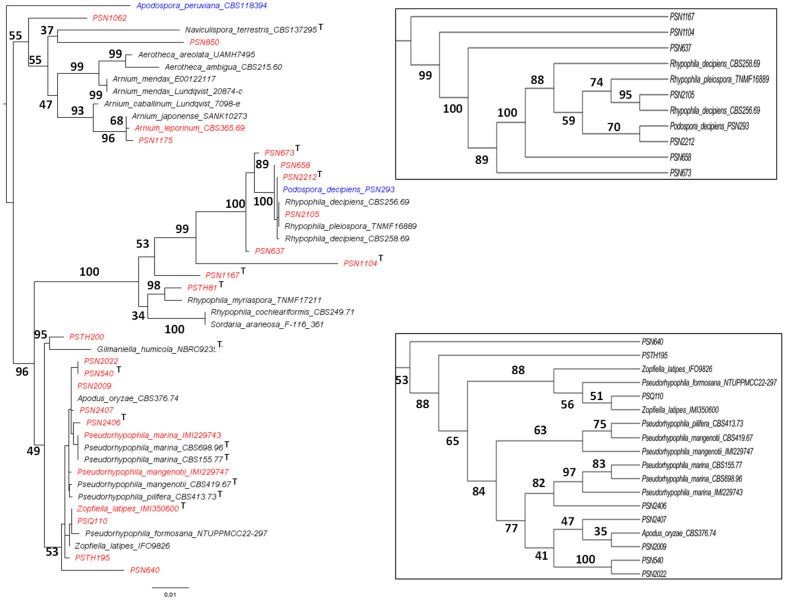
ITS + LSU phylogenetic tree of the *Naviculisporaceae*. The tree was rooted with *Apodospora peruviana* CBS 118,394. The strains whose genome sequences were obtained in the present study are shown in red, and the strains whose genome sequences were previously published are in blue [14]. On the left panel, Ts indicate ex-type strains. Insets show cladograms of *Rhypophila* and *Pseudorhypophila* displaying bootstrap values.

**Figure 2:**
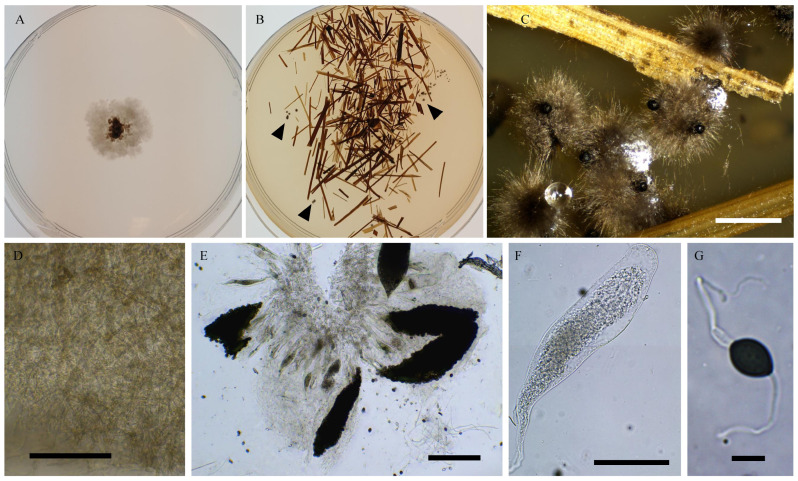
Morphology of *Podospora aff. araneosa* PSN1062. Cultures on M2 (**A**) and M0 + hay (**B**); arrowheads point towards perithecia. Both plates were inoculated at the centre and incubated for one month at 27 °C and 24 h illumination. Growth was very slow on M2 and much faster on M0 + hay. Perithecia differentiated only on M0 + hay after 2–4 weeks at room temperature (**C**). Their diameter measured 935 ± 86 µm (*n* = 10). Their peridium was light brown (**D**) and densely covered with long flexuous hair (**C**). They contained rosettes of clavate asci bearing around 256 ascospores (**E**). Young asci (**F**) were at first filled with ascospores only in the central part. Ascospores were bicellular (**G**) and multiseriate (**E**,**F**). Spore heads were 19.8 ± 1.2 × 13.9 ± 0.5 µm (*n* = 30), and olivaceous when young and dark brown when mature (**G**). The primary appendages were 9.5 ± 1.6 × 4.4 ± 0.9 µm (*n* = 10), and cylindrical and hyaline (**G**). Two thin and long secondary appendages of variable length were present, one at the apex of the ascospore and one at the end of the primary appendage (**G**). After ejection, the primary and secondary appendages were often lost. Except for the slightly larger sizes of the perithecia and slightly smaller size of the ascospores, this description fits well with that of *P. araneosa* (Cain) Cain. Scale bars: **C** = 1 mm, **D**,**F** = 100 µm, **E** = 200 µm, and **G** = 10 µm.

Nine strains, namely PSN1062, PSN850, PSTH81, PSN1167, PSN1104, PSN637, PSN673, PSTH200, and PSN640, were distant from previously sequenced species in the ITS + LSU tree (Figure 1), indicating that they belonged to separate and possibly new species. We were able to obtain fruiting in vitro for five of them: PSN1062, PSTH81, PSN1167, PSN1104, and PSN673. Morphological analysis (Figure 2) showed that four of these strains produced sexual structures with distinct and previously undescribed morphologies, making them four species new to science (see Section 3.4), while PSN1062 produced perithecia similar to those of *Podospora araneosa* (Cain) Cain [34,35].

PSN1062 ITS had 15 differences out of 334 aligned nucleotides with the ITS of *P. araneosa* F-116,361, which was used in previous phylogenetic analyses of the *Naviculisporaceae* (see accession number in Table 2). Accordingly, both strains branched at different positions in the ITS + LSU tree (Figure 1). PSN1062 also had four differences out of 362 aligned nucleotides in the ITS with *P. araneosa* TNM F17207 (GenBank accession number EF197073.1) and 36 differences in the *gpd* gene out of 492 aligned nucleotides (GenBank accession number EF197097.1). It did not contain the secondary metabolite gene cluster of *Sordaria araneosa* ATCC 36,386 involved in sordarin biosynthesis (*S. araneosa* is the basionym of *P. araneosa*; GenBank accession number LC079035.1). However, it did contain a related cluster, i.e., containing similar genes, albeit with low sequence similarity between the two strains. Overall, these data showed that the *P. araneosa* strains previously investigated likely belong to at least two different species and that PSN1062 belongs to a third one. Moreover, PSN1062 perithecia were slightly larger than those of the original *P. araneosa* description (diameter = 935 ± 86 µm, instead of 550–700 × 400–460 µm) and the ascospores were slightly smaller (spore head = 19.8 ± 1.2 × 13.9 ± 0.5 µm, instead of 24–26 × 16–17 µm; primary appendage = 9.5 ± 1.6 × 4.4 ± 0.9 µm, instead of 11–12 × 6–8 µm), making it unclear whether it was a bona fide *P. araneosa*. This strain was thus labelled as *P. aff. araneosa*, pending the determination of DNA sequences for the type of *P. araneosa* TRTC 5211, to allow for the accurate delimitation of *P. araneosa*.

The four strains for which we could not obtain fruiting in vitro (PSN850, PSN637, PSTH200 and PSN640) are not formally described here, but may belong to four new species. PSN850 was related to *N. terrestris* Stchigel, Y. Marín, Cano and Guarro and likely belonged to the same genus; it was thus named *Naviculispora* sp. Similarly, PSN637 was related to *R. myriaspora* (P. Crouan and H. Crouan) Y. Marín, A.N. Mill. And Guarro and was thus named *Rhypophila* sp. PSTH200 was related to *G. humicola* G.L. Barron and was named *Gilmaniella* sp. and PSN640 was related to *P. mangenotii* (Arx and Hennebert) Y. Marín and Stchigel and was thus named *Pseudorhypophila* sp.

**Figure 3:**
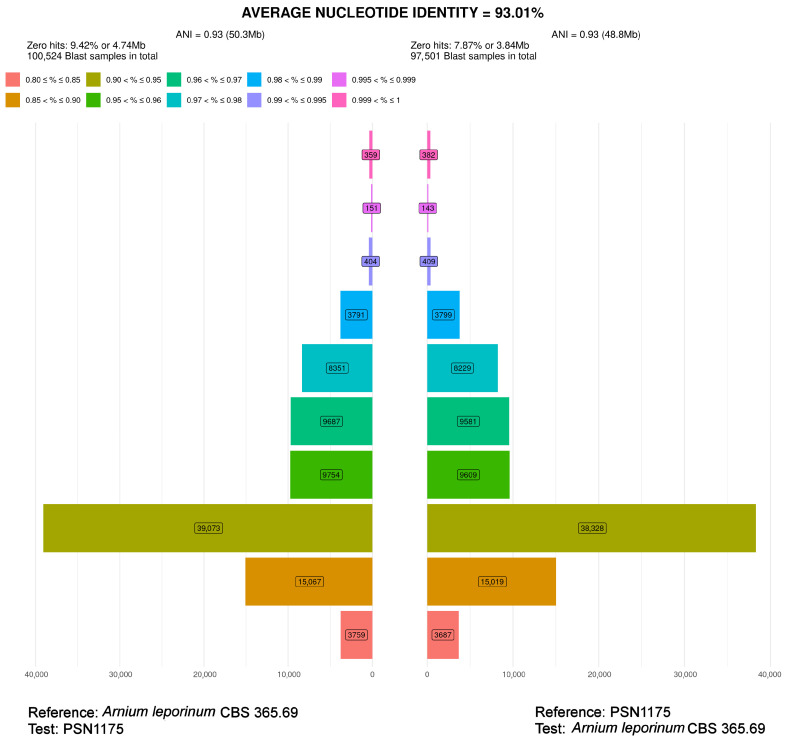
Average nucleotide identity (ANI) analysis of PSN1175 and *Arnium leporinum* CBS 365.69. Horizontal bars quantify the number of BLAST results for each range of similarity percentages defined and colour-coded at the top of the plot. The calculations were made in both directions, i.e., with PSN1175 as query (test) on the left and CBS 365.69 as subject (reference) and reciprocally on the right. The final ANI is the average of the two values. The numbers of BLAST hits obtained for each percentage range are boxed in the corresponding bars and the total number of BLAST searches made during the analysis is given at the top of the figure. The zero hits correspond to BLASTs where no similarity was found. For more information, see ref. [2]. PSN1175 and CBS 365.69 displayed very few sequences with a similarity higher than 99.5%, corresponding to the pink and magenta bars at the top of the graphic. Moreover, each strain had about 8% to 9% of specific sequences.

PSN1175 clustered with *A. leporinum* CBS 365.69 and *A. japonense* SANK10273 (Figure 1). ANI analysis indicated that PSN1175 and *A. leporinum* CBS 365.69 represented distinct species, as their genomes were only 93.01% similar and each strain had 8 to 9% of specific sequences (Figure 3). PSN1175 displayed no difference with *A. japonense* SANK10273 across 1359 aligned nucleotides of the LSU sequence and five differences across 670 aligned nucleotides for *TUB2*, leaving its species status unresolved. As PSN1175 did not produce perithecia in vitro, its identification as *A. japonense* Furuya and Udagawa could not be verified. It is therefore provisionally designated *A. aff. japonense*.

Strains PSN658, PSN2212 and PSN2105 clustered in the ITS + LSU tree with the previously sequenced PSN293 as well as with *R. decipiens* CBS 258.69, *R. pleiospora* TNMF16889 and *R. decipiens* CBS 256.69 (Figure 1). As shown in Table 3, the ANI values of pairwise comparisons between the genomes of PSN658, PSN2212, PSN2105, CBS 256.69 and PSN293 were much lower than 99%, except for PSN2105 and CBS 256.69, indicating that the strains belonged to different species, except PSN2105 and CBS 256.69.

**Figure 4:**
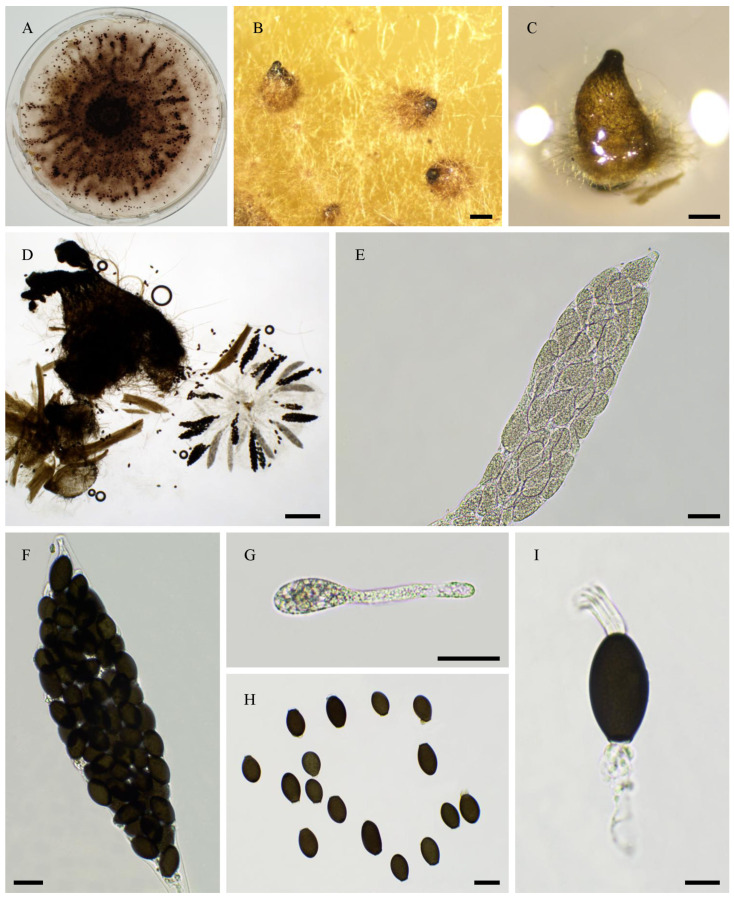
Morphology of *Rhypophila myriaspora* PSN2105. Mycelium on M2 medium after two weeks of growth (**A**). Perithecia were readily differentiated on M0 + miscanthus after 2–3 weeks incubation at 27 °C with constant illumination; they were 542 ± 68 µm diam (*n* = 10), pyriform, and covered with numerous hairs (**B**); their neck was black and slightly elongated (**B**–**D**). Their peridium was pale brown and semi-translucid (**C**). Asci were 64-spored, clavate with a tapering and narrow apex (**E**,**F**). Ascospores were multiseriate and bicellular (**F**–**I**). Ascospore heads were 27.3 ± 2.8 × 17.1 ± 1.2 µm (*n* = 30), ellipsoidal with a slightly flattened base, which was hyaline at first (**E**,**G**), then green and black when matured (**F**,**H**,**I**). The primary appendage was long (often longer than the spore head) and flat (**G**), frequently collapsing (**G**–**I**), 38.6 ± 5.2 × 6.3 ± 0.9 µm (*n* = 10). Secondary appendages were present at the apex of the spore head as a fan-shaped structure of four fibrillar strands and at the junction between the spore head (**I**). Primary and secondary appendages were often lost upon ascospore ejection. This description fits with the ones made by Lundqvist for *R. myriaspora* (perithecium = 670–1150 × 400–530 µm, spore head = 23–34 × 14–19 µm, primary appendage = 18–45 × 14–19 µm; [16]) and Doveri (perithecium = 625–750 × 400–500 µm, spore head = 27.3–34.6 × 17.8–19 µm, primary appendage = 30–37 × 5–7 µm; [36]). Scale bars: **B**–**D** = 200 µm; **E**–**H** = 20 µm; **I** = 10 µm.

CBS 256.69 is labelled as *R. decipiens* in the Westerdjik Institute collection. However, PSN2105 had an ITS sequence identical to that of *R. myriaspora* CBS 115,804 (GenBank accession number DQ166961.1) and produced 64-spored asci typical of *R. myriaspora* (P. Crouan and H. Crouan) Y. Marín, A.N. Mill. And Guarro (Figure 4). Unfortunately, CBS 256.69 did not fructify in vitro, making it impossible to confirm species assignment by morphological analysis; however, the genome sequence indicated that it is most likely a *R. myriaspora* strain, and it is designated as such hereafter.

**Figure 5:**
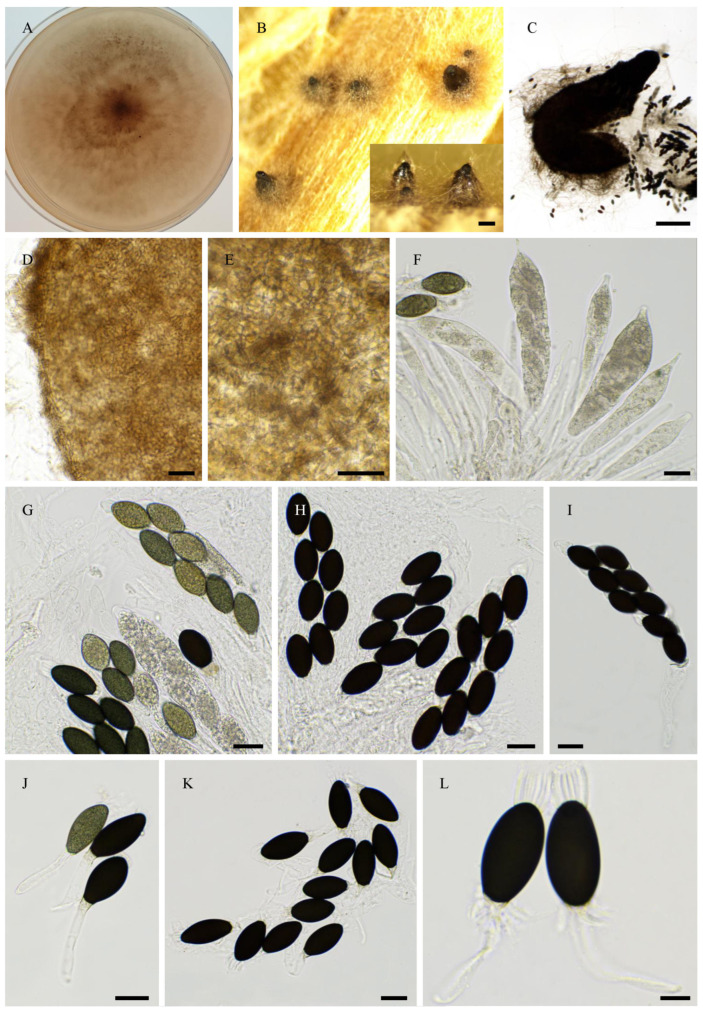
Morphology of *Rhypophila decipiens* PSN293. Mycelium on M2 medium after two weeks of growth (**A**). Perithecia were readily differentiated on M0 + miscanthus after 2–3 weeks incubation at 27 °C with constant illumination; they had a diameter of 439 ± 71 µm and were pyriform and covered with numerous hairs (**B**); their neck was black and slightly elongated (**B**,**C**). Their peridium was pale brown and semi-translucid; their *textura* was *angularis* (**D**,**E**). Asci were eight-spored, clavate with a tapering and narrow apex (**F**,**G**). Ascospores were biseriate and bicellular (**G**–**L**). Ascospore heads were 35.5 ± 2.8 × 18.6 ± 1.2 µm (*n* = 30), ellipsoidal with a slightly flattened base, and hyaline at first (**F**,**G**), then green (**G**) and black when matured (**H**–**L**). The primary appendage was very long (often longer than the spore head) and flat (**J**), frequently collapsing (**L**), 44.3 ± 7.3 × 6.5 ± 1.5 µm (*n* = 10). Secondary appendages were present at the apex of the spore head as a fan-shaped structure of juxtaposed fibrils and at the junction between the spore head and the primary appendage as fibrils often two in number (**L**). This description fits with the ones made for *R. decipiens* by Mirza and Cain (perithecium = 600–800 × 350–450 µm, spore head = 35–46 × 19–23 µm, primary appendage = 40–60 × 7–8 µm; [35]), Lundqvist (perithecium = 480–1050 × 290–530 µm, spore head = 36–42 × 20–22 µm, primary appendage = 55–73 × 7.2–8.5 µm; [16]), and Doveri (perithecium = 750–1050 × 620–740 µm, spore head = 34.6–42 × 19–23 µm, primary appendage = 33–42 × 5.5–8 µm; [36]). Scale bars: **B** = 200 µm; **C** = 200 µm; **D**–**K** = 20 µm; **L** = 10 µm.

PSN293 was closely related, in terms of nucleotide identity, to *R. decipiens* CBS 258.69 used in previous analyses of the *Naviculisporaceae*: we observed no difference in LSU across 1008 aligned nucleotides, one difference across 391 aligned nucleotides in the ITS sequence, three differences across 1198 aligned nucleotides in *RPB2* (GenBank accession number AY780187.1), and no difference in *TUB2* across 969 aligned nucleotides (GenBank accession number AY780130.1). Moreover, The ITS sequence of PSN293 was identical to that of *R. decipiens* CBS 113,104 across 559 aligned nucleotides (GenBank accession number AY515359.1). It produced eight-spored asci typical of *R. decipiens* (G. Winter ex Fuckel) Y. Marín, A.N. Mill. And Guarro confirming that this strain actually belongs to this species (Figure 5).

**Figure 6:**
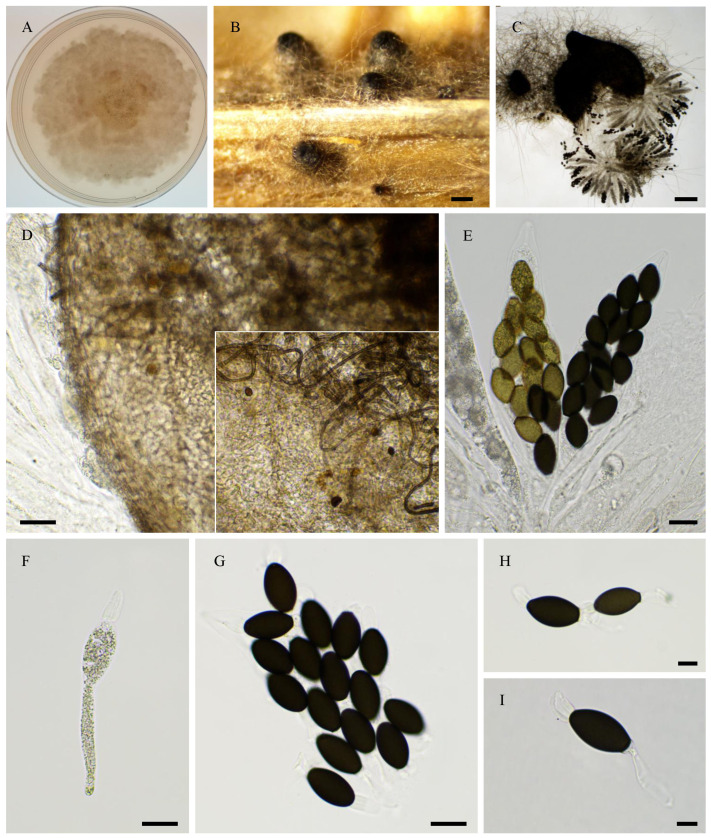
Morphology of *Rhypophila pleiospora* PSN658. Mycelium on M2 medium after two weeks of growth (**A**). Perithecia were readily differentiated on M0 + miscanthus after 2–3 weeks incubation at 27 °C with constant illumination; they were 579 ± 71 µm diam (*n* = 10), pyriform and covered with numerous hairs (**B**); their neck was black and short (**B**,**C**). Their peridium was pale brown and semi-translucid, their *textura* was *angularis* (**D**). Asci were sixteen-spored, clavate with a tapering and narrow apex (**E**). Ascospores were biseriate or triseriate and bicellular (**E**–**I**). Ascospore heads were 29.8 ± 2.3 × 17.0 ± 1.2 µm (*n* = 30), ellipsoidal with a slightly flattened base, and hyaline at first (**E**,**F**), then green (**E**) and black when matured (**G**–**I**). The primary appendage was long (often longer than the spore head) and flat (**F**), frequently collapsing (**G**–**I**), 38.7 ±.9.6 × 7.1 ± 1.5 µm (*n* = 10). Secondary appendages were present at the apex of the spore head as a fan-shaped structure of juxtaposed fibrils and at the junction between the spore head and the primary appendage as fibrils often two in number (I). This description fits with the ones for *R. pleiospora* made by Mirza and Cain (perithecium = 600–1000 × 375–550 µm, spore head = 31–36 × 19–24 µm for 16-spored asci, primary appendage = 25–50 × 5–8 µm; [35]), Lundqvist (perithecium = 530–1100 × 335–550 µm, spore head = 30–37 × 18–23 µm, primary appendage = 35–65 µm; [16]), and Doveri (perithecium = 1000–1200 × 500–650 µm, spore head = 31.5–36.7 × 17.8–21 µm, primary appendage = 22–45 × 5–7 µm; [36]). Scale bars: **B**,**C** = 200 µm; **D**–**G** = 20 µm; **H**,**I** = 10 µm.

Intriguingly, *R. pleiospora* TNM F16889, used in a previous phylogenetic analysis of the *Naviculisporaceae*, had four differences in the ITS across 544 aligned nucleotides with *R. pleiospora* CBS 113,107 (GenBank accession number AY515364.1) and four differences across 550 aligned nucleotides with PSN658. These results suggested that these strains were unlikely to belong to the same species. Moreover, PSN658 had an ITS sequence strictly identical to that of *R. pleiospora* CBS 113,107 across 556 aligned nucleotides. PSN658 also produced the 16-spored asci typical of *R. pleiospora* (G. Winter) Y. Marín, A.N. Mill. And Guarro (Figure 6). This suggested that the TNM F16889 strain used in previous phylogenetic analysis may not belong to *R. pleiospora*, but is to another related species. Finally, PSN2212 produced eight-spored asci with ascospores that were larger than those of typical *R. decipiens*, making it a species new to science (see Section 3.4).

**Figure 7:**
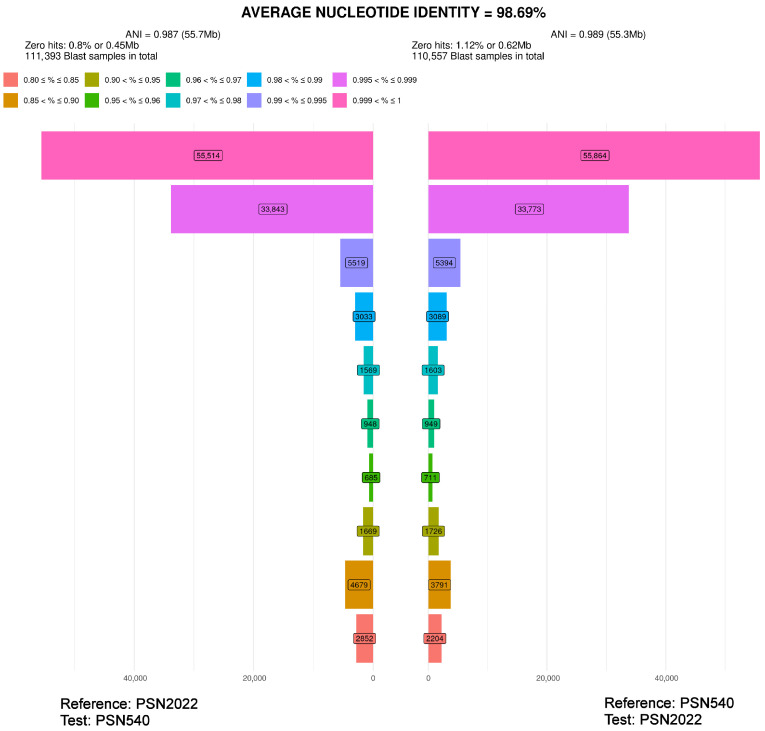
ANI analysis of PSN540 and PSN2022. See legend of Figure 3 for a detailed explanation of the graphic. The analysis revealed that the two strains shared mostly highly similar sequences (e.g., with a similarity higher than 99.5%; pink and magenta bars). They have very few specific sequences (around 1%). The ANI is lowered to less than 99% due to the presence of about 4% of very dissimilar sequences with a similarity of around 85 and 90% (bars around the yellow-orange ones at the bottom of the graphic).

Strains PSTH195, PSQ110, PSN2406, PSN540, PSN2022, PSN2407 and PSN2009 clustered with *Zopfiella latipes* IMI350600 and IFO9826, *P. mangenotii* IMI229747 and CBS419.67, *P. pilifera* CBS 413.73, *P. marina* IMI229743, CBS 155.77 and CBS 698.96, *P. formosana* NTUPPMCC 22-297, and *Apodus oryzae* CBS 376.74 (Figure 1). ANI analyses for these strains (Table 4) yielded values much lower than 99%, except for PSQ110/IMI350600 and PSN540/PSN 2022. In the latter case, the ANI value was lower than 99%, but this was due to the presence in the genome of a substantial proportion (approximately 4%) of highly divergent sequences with a similarity of 85% to 90% (Figure 7). The two strains shared mostly very similar sequences with a percentage of identity higher than 99.5% and contained few specific sequences (about 1% of their genome), indicating that they belonged to the same species (Figure 7).

**Figure 8:**
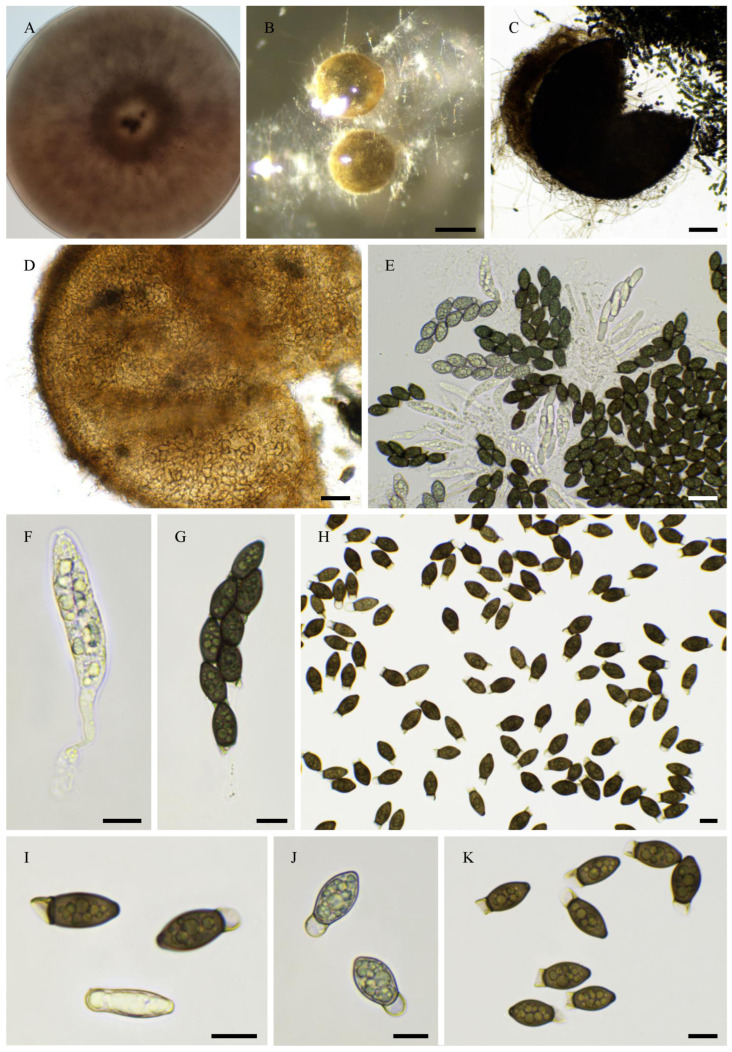
Morphology of *Pseudorhypophila latipes* comb. nov. IMI350600. Mycelium on M2 medium after two weeks of growth (**A**). Perithecia, non-ostiolate, were readily differentiated on M2 and M0 + miscanthus after 2–3 weeks incubation at 27 °C with constant illumination, superficial or immersed, black, globose to subglobose, and they were 449 ± 89 µm diam (*n* = 10) (**B**,**C**); their peridium was pale brown and semi-translucid, tissue type was *textura angularis* (**D**). Asci were eight-spored, and clavate with a tapering and narrow apex (**E**–**G**). Ascospores were biseriate and bicellular (**G**). Ascospore heads were 18.0 ± 0.8 × 10.6 ± 0.5 µm (*n* = 30), ovoid with a slightly flattened base, and hyaline at first (**I**), then green (**J**) and brown to dark brown when matured (**H**,**I**,**K**). The primary appendage was a rounded head and hyaline (**J**), frequently collapsing at maturity (**K**), they were 4.5 ± 0.6 × 6.4 ± 0.6 µm (*n* = 10). This description fits the original description of Lundqvist (as *Tripterospora latipes:* perithecium = 290–700 µm diam, spore head = 15.5–20.5 × 10–13 µm, primary appendage = 6.3–7.7 × 5.5–7 µm; [38]). Scale bars: **B** = 200 µm; **C** = 100 µm; **D**,**E** = 20 µm; **F**–**K** = 10 µm.

Morphological analyses confirmed that IMI350600 and PSQ110 belong to *Z. latipes* (N. Lundq.) Malloch and Cain (Figure 8 and Figure S1), as expected for IMI350600 since it is the ex-type strain for this species. Since *Z. latipes* clearly branched with the known *Pseudorhypophila* species in the ITS and LSU tree (Figure 1), it was renamed as *P. latipes* (N. Lundq.) Tangthirasunun and Silar comb. nov. Note that IMI350600 and PSQ110 showed no differences across 559 aligned nucleotides in their ITS compared to *Z. latipes* IFO9826 previously used in a phylogenetic analysis of *Zopfiella* and allied genera [37]. There were two differences across 842 aligned nucleotides in their LSU and seven differences in *TUB2* across 491 aligned nucleotides (accession number AY999146.1), suggesting that these strains do not belong to the same species, especially *P. formosana*, a recently identified species [13], branched in the ITS + LSU tree between IFO9826 and IMI350600/PSQ110 (Figure 1).

**Figure 9:**
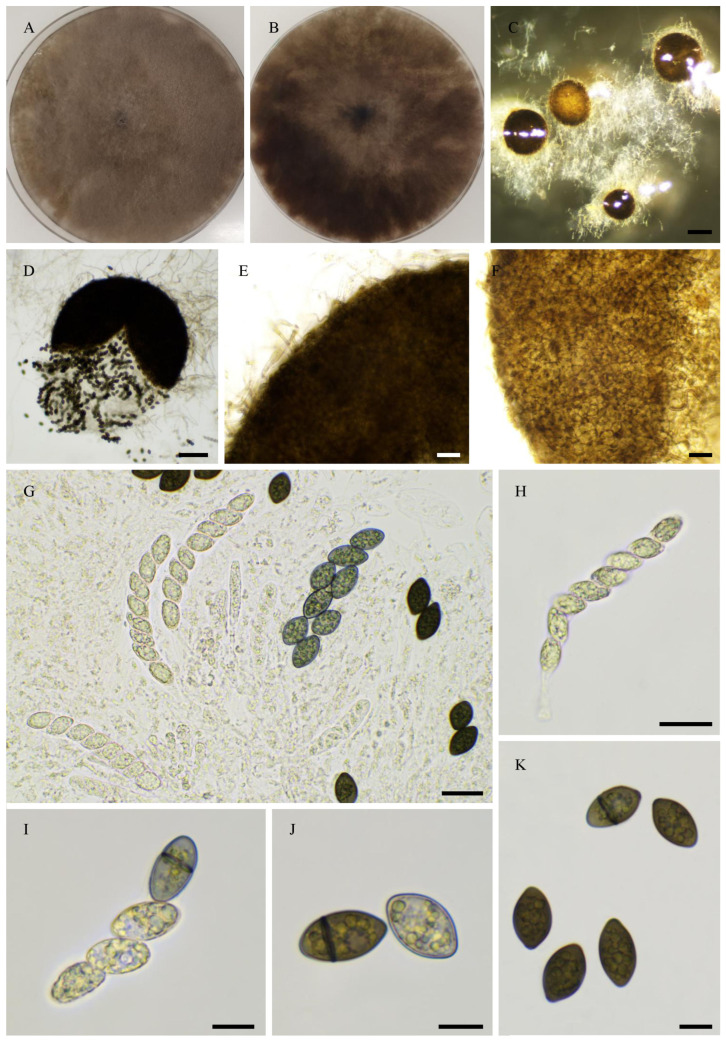
Morphology of *Pseudorhypophila oryzae* PSN2009. Mycelium on M2 medium after two weeks of growth (**A**,**B**). Non-ostiolate perithecia were readily differentiated on M0 + miscanthus after 2–3 weeks incubation at 27 °C with constant illumination; pyriform, superficial or semi-immersed, 415 ± 107 µm diam (*n* = 10) (**C**,**D**). Their peridium was pale brown and semi-translucid, and tissue type was *textura angularis*. (**E**,**F**). Paraphyses absent. Asci were eight-spored, clavate with a tapering and narrow apex (**G**). Ascospores were uniseriate in young asci and were disordered in older ones (**G**,**H**). Ascospore heads were 21.7 ± 2.2 × 13.2 ± 1.1 μm (*n* = 30), ellipsoidal to oval, and hyaline at first, then green and brown to dark brown when matured (**I**–**K**). Few ascospores (less than 10%) were 1-septate (septum slightly curved or straight toward the apex), with one cell 12.4 ± 1.9 µm and the other 8.5 ± 1.8 μm (*n* = 15) (**I**–**K**). This description fits with that of *A. oryzae* Carolis and Arx (non-ostiolate perithecium = 200–500 µm; ascospore = 21–27 × 10–14 µm, more than 90% of the ascospores remaining single-celled; [40]). Scale bars: **C** = 200 µm; **D** = 100 µm; **E**–**H**= 20 µm; **I**–**K** = 10 µm.

PSN540, PSN2022, PSN2009 and PSN2407 branched with CBS 376.74, the ex-type strain of *A. oryzae* (Figure 1). PSN540 and PSN2022 had three differences out of 550 aligned nucleotides in their ITS sequence with *A. oryzae* CBS 376.74, no differences out of 839 aligned nucleotides in their LSU region, and three differences out of 492 aligned nucleotides in *TUB2* (GenBank accession num AY681234.1). PSN2407 displayed no difference with *A. oryzae* CBS 376.74 in the ITS across 550 aligned nucleotides, one difference across 839 aligned nucleotides in the LSU region, and seven differences across 492 aligned nucleotides in *TUB2*. PSN2009 had zero differences with *A. oryzae* CBS 376.74 in the ITS and LSU regions and a single difference out of 492 aligned nucleotides in *TUB2*. In line with its close proximity to *A. oryzae* CBS 376.74 based on sequence comparisons, morphological analysis confirmed that PSN2009 exhibited a morphology typical of *A. oryzae* (Figure 9). Hence, PSN2009 was assigned to *A. oryzae*, while PSN540 and PSN2022 belonged to a closely related species (see Section 3.4) and PSN2407 was assigned to another separate new species. Unfortunately, PSN2407 did not fructify in vitro, preventing us from assessing whether it was a species new to science or not. It was thus provisionally called *Pseudorhypophila* sp. *A. oryzae* was renamed as *P. oryzae* (Carolis and Arx) Tangthirasunun and Silar comb. nov., owing to its branching with other *Pseudorypophila* species in the ITS + LSU tree. Note that *A. deciduus* strain CBS 506.70, belonging to the type species for the *Apodus* genus, Malloch and Cain, is in the *Schizotheciaceae* [39], a position confirmed by whole genome sequence analysis (unpublished data).

**Figure 10:**
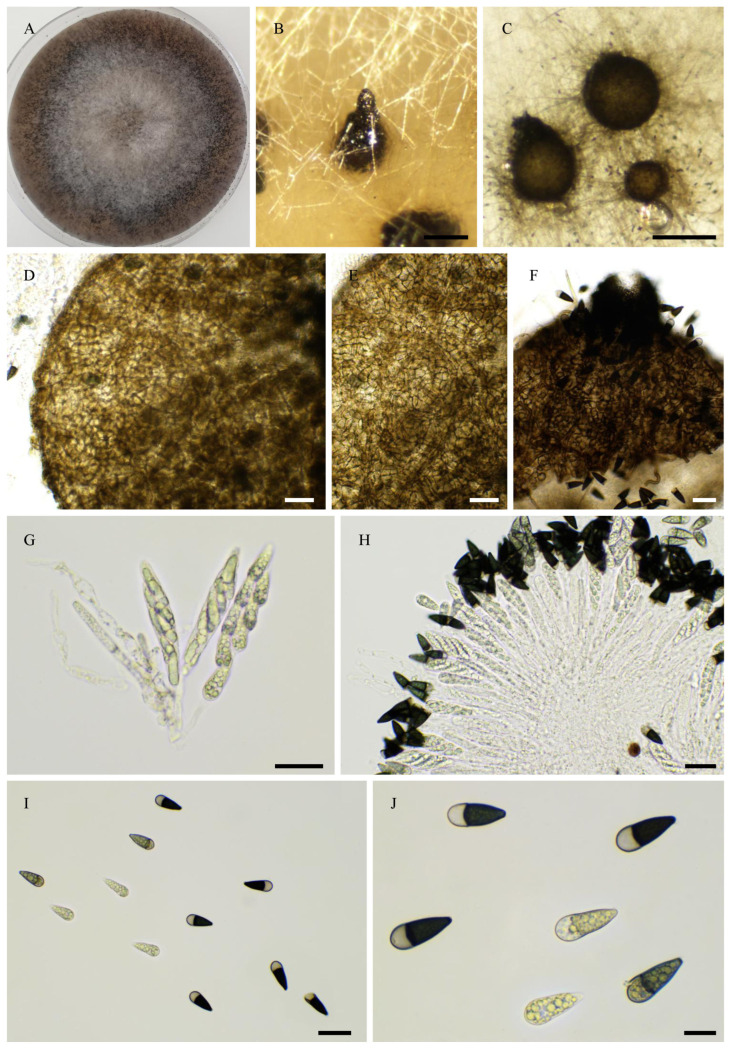
Morphology of *Pseudorhypophila mangenotii* IMI229747. Mycelium on OA medium after two weeks of growth (**A**). Perithecia were readily differentiated on OA after 2–3 weeks incubation at 27 °C with constant illumination, immersed or semi-immersed, black; they were 266 ± 47 µm diam (*n* = 10) (**B**,**C**), with a clearly defined and narrow neck (**B**,**F**). Their peridium was pale brown and semi-translucid, and tissue type was *textura angularis* (**D**,**E**). Asci were eight-spored, clavate with a tapering and narrow apex (**G**,**H**). Ascospores were biseriate and bicellular (**G**–**I**). Ascospore heads were 15.8 ± 0.9 × 8.0 ± 0.4 µm (height × base size; *n* = 30), oval with a slightly flattened base, and hyaline at first (**G**,**I**,**J**), then green (**I**) and black when matured (**I**,**J**). The primary appendage shape had a rounded end and was hyaline (**I**,**J**), and they were 5.7 ± 0.5 × 7.9 ± 0.4 µm (*n* = 10). This description fits the original one made by von Arx and Hennebert (as *Triangularia mangenotii*: perithecium = 170–260 µm diam., ascospore size = 21–24 × 7–9 µm, primary appendage = 7–9 µm diam.; [41]). Scale bars: **B**,**C**= 200 µm; **D**–**I** = 20 µm; **J** = 10 µm.

**Figure 11:**
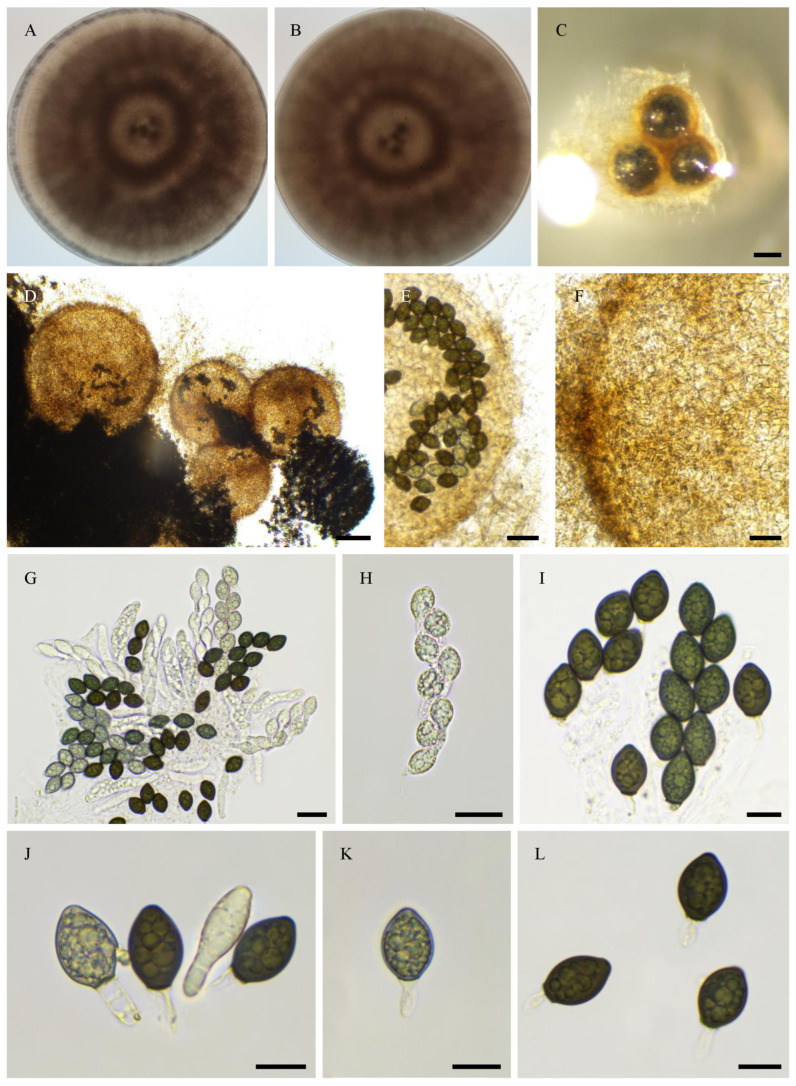
Morphology of *Pseudorhypophila marina* IMI229743. Mycelium on medium after two weeks of growth (**A**,**B**). Perithecia, non-ostiolate, were readily differentiated on M2, M0 + miscanthus, OA, V8 and M0 + hay after 2–3 weeks incubation at 27 °C with constant illumination, superficial or immersed, black, globose to subglobose, and they were 287 ± 20 µm diam (*n* = 10) (**C**,**D**). Their peridium was pale brown and semi-translucid, tissue type was *textura angularis* (**E**,**F**). Asci were eight-spored, clavate with a tapering and narrow apex (**G**,**H**). Ascospores were biseriate and bicellular (**G**–**I**). Ascospore heads were 17.7 ± 0.9 × 12.2 ± 0.5 µm (*n* = 30), oval with a slightly flattened base or somewhat truncate base, and hyaline at first (**G**,**H**,**J**), then green (**J**,**K**) and brown to dark brown when matured (**J**,**L**). The primary appendage was semi-long and flat, hyaline, frequently collapsing (**J**–**L**), and they were 12.2 ± 4 × 5.2 ± 1.3 (*n* = 10). IMI229743 (=NHL 2731) is the ex-type strain for *P. marina* and this description fits the original one made by Furuya and Udagawa (as *Zopfiella marina*: perithecium = 180–450 µm, ascospore head = (14–)15–20 × 10–13(–14) µm, primary appendage = 8–10 × 3–4 µm; [42]). Scale bars: **C**,**D** = 100 µm; **E**–**H** = 20 µm; **I**–**L** = 10 µm.

Morphological analysis confirmed that IMI229747 and IMI229743 belonged to *P. mangenotii* and *P. marina*, respectively (Figure 10 and Figure 11). Consistent with this assignment, IMI229747 showed no difference compared to *P. mangenotii* CBS 419.67 across 891 aligned nucleotides in the LSU region, no difference across 484 aligned nucleotides in the ITS, no difference across 618 aligned nucleotides in *TUB2* (GenBank accession num KP981571.1), and one difference across 1022 aligned nucleotides in *RPB2* (GenBank accession num KP981627.1). Similarly, IMI229743 showed no difference with *P. marina* CBS 155.77 out of 1052 aligned nucleotides in its ITS + LSU region and no difference out of 849 aligned nucleotides in *RPB2* (GenBank accession number MK876813.1); it also showed with *P. marina* CBS 698.96 no difference out of 1052 aligned nucleotide in the ITS + LSU region and one difference out of 831 aligned nucleotides in *RPB2* (GenBank accession number MK876815.1; the species is labelled as *Zopfiella submersa* in GenBank).

Morphological analysis also showed that PSN2406 is a new species (see Section 3.4). Finally, PSTH195 did not fructify in vitro making it difficult to determine whether it was a species new to science. It was thus provisionally named *Pseudorhypophila* sp., as it appeared closely related to the other *Pseudorhypophila* species.

Overall, we generated complete genome sequences for the following:

(1) Six previously known species, two of which are newly placed in the *Naviculisporaceae*: *R. myriaspora* (PSN2105 and likely CBS 256.69), *R. pleiospora* (PSN658), *P. latipes* (formerly *Z. latipes*; IMI350600 and PSQ110), *P. mangenotii* (IMI229747), *P. marina* (IMI229743) and *P. oryzae* (formerly *A. oryzae*; PSN2009), adding to the previously sequenced *R. decipiens* (PSN293);
(2) Seven new species, including five *Rhypophila* species: *R. thailandica* sp. nov. (PSTH81), *R. reunionensis* sp. nov. (PSN1167), *R. brasiliensis* sp. nov. (PSN1104), *R. camarguensis* sp. nov. (PSN673) and *R. alpibus* sp. nov. (PSN2212), as well as two *Pseudorhypophila* ones: *P. guyanensis* sp. nov (PSN2406), and *P. gallica* sp. nov. (PSN540 and PSN2022);
(3) Eight newly isolated strains awaiting further characterization, some of which may belong to species new to science: *P. aff. araneosa* PSN1062, *Naviculispora* sp. PSN850, *A. aff. japonense* PSN1175, *Rhypophila* sp. PSN637, *Gilmaniella* sp. PSTH200, *Pseudorhypophila* sp. PSN640, *Pseudorhypophila* sp. PSN2407 and *Pseudorhypophila* sp. PSTH195;
(4) One collection strain: *A. leporinum*: CBS 365.69.

### 3.2. Phylogenomic Analysis

**Figure 12:**
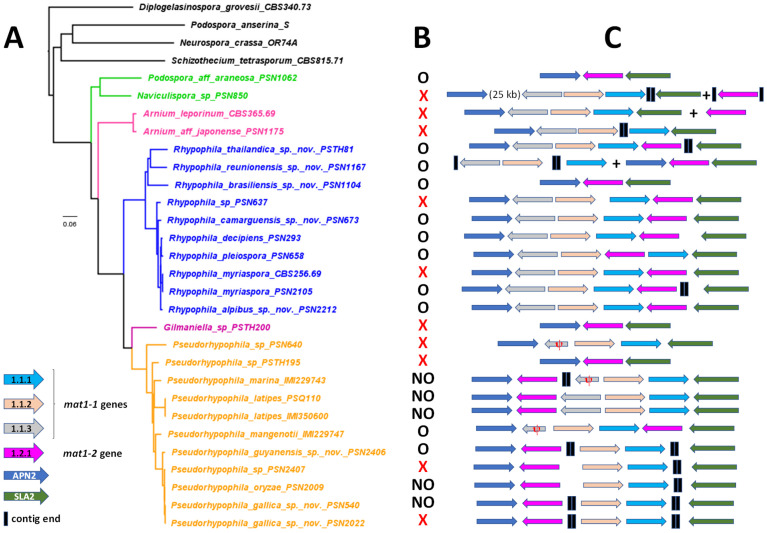
Phylogenomic tree (**A**) in vitro fertility (**B**) and mating-type locus organizations (**C**) of the sequenced *Naviculisporaceae*. Presence of fruiting bodies is recorded as ostiolate perithecia (O) non-ostiolate perithecia (NO); red cross: no in vitro fruiting. For the schematic representations of the mating-type loci, the black vertical bar indicates the end of contigs, often due to the presence of repeated sequences. The red ψ denotes probable *mat1-1-3* pseudogenes.

As shown in Figure 1, statistical supports for many nodes in the ITS + LSU tree were very low. We thus computed a phylogenomic tree using the concatenation of sequences of 2798 BUSCO genes. We used as outgroups some representatives of major families of *Sordariales* (*Podospora anserina* for the *Podosporaceae*, *Neurospora crassa* for the *Sordariaceae* and *Shizothecium tetrasporum* for the *Schizotheciaceae*), as well as *Diplogelasinospora grovesii*. This later species belongs to the *Diplogelasinosporaceae*, the most divergent family of the *Sordariales* [14] and was used to root the tree (Figure 12A). All nodes received strong support, with 100% bootstrap values. As in the ITS + LSU tree, *Pseudorhypophila* and *Rhypophila* were each recovered as monophyletic genera. However, the relationships between the different species within each genus were distinct from those in the ITS + LSU tree. Additionally, *Gilmaniella* sp. PSTH200 branched between these two genera, closer to *Pseudorhypophila*, in both trees. Yet, the branch length in the phylogenomic tree is short, making it unclear whether it was a genus distinct from *Pseudorhypophila*. The two *Arnium* species clearly defined a separate genus, whose naming will await the molecular characterization of *A. lanuginosum* Nitschke; the type species for the genus. *P. aff. araneoase* PSN1062 and *Naviculispora* sp. PSN850 were sisters to all the other strains.

A noticeable difference between the *Rhypophila* and *Pseudorhypophila* species is their genome sizes: between 50 and 56 Mb for *Pseudorhypophila*, except for the most divergent strain of the genus (PSN640, with 47.5 Mb), and between 45 and 50 Mb for *Rhypophila* species (Table 1 and Table S1). Overall, all *Naviculisporaceae* species tended to have larger genome sizes exceeding 45 Mb, compared to the other *Sordariales*, which typically had genome sizes ranging from 35 to 40 Mb.

As shown in Table 1, the *Rhypophila* species were mostly recovered from dung, while the *Pseudorhypophila* ones were isolated from soils. The former differentiated ostiolate perithecia can eject their ascospores and the latter mostly non-ostiolate ones cannot eject their ascospores. This is consistent with the hypothesis that dispersing their ascospores over long distances from the growing substrate may be advantageous to dung-inhabiting fungi, while soil fungi ascospores might only be needed to persist within the soil. Therefore, they may not benefit from actively dispersing their ascospores, as this could be an energy-consuming process.

### 3.3. Mating-Type Locus Analysis

As shown in Figure 12B, some strains did not fructify in vitro under the tested conditions (i.e., at 27 °C, with 24 h of light and on five different media: M2, Oat Meal Agar, V8, M0 + miscanthus and M0 + hay). One possibility was that these strains were heterothallic and that the recovered isolates carried a single mating type, although complete asci or batches of several ascospores were used to initiate cultures. To test this possibility, mating-type loci were manually annotated (Figure 12C), and putative breeding systems were inferred from the structures of the loci (Figure 12C). Three kinds of mating type-locus structures were observed.

Firstly, most strains (17 out of 26) contained both the genes for the *mat1-1* idiomorph (*mat1-1-1*, *mat1-1-2* and *mat1-1-3*) and the *mat1-2-1* gene of the *mat1-2* idiomorph, clustered together and flanked by the *APN2* gene on one side and the *SLA2* gene on the other (Figure 12C). Note that, in some cases, although the locus was split onto different contigs, the complete locus could nonetheless be reassembled because the *mat* genes were located at the edges of contigs. These thus contained the mating-type locus previously described for homothallic *Sordariales* such as *Sordaria macrospora* [43,44,45]. Most of these strains readily fructified in vitro. The exceptions were CBS 356.69, which had been kept in vitro for a long time, PSN2022, which fructified in an unreproducible manner, as well as PSN637 and PSN2407. These results altogether suggested that these strains were likely homothallic, and that the three, possibly four, later required special conditions to fructify, not met in vitro.

Secondly, three strains (PSN850, CBS 365.69 and PSN1167) contained the two idiomorphs on different contigs, with a single one bordered by *APN2* and *SLA2 *(Figure 12C). Such structure could be due to a unusual homothallic mating-type locus structure in which the two idiomorphs would not be linked, or to heterothallism. Indeed, because we sequenced putative *mat1-1/mat1-2* heterokaryons, one would expect the two idiomorphs to be on different contigs, each bordered by *APN2* and *SLA2*. However, assembly programmes do not perform well on such heterozygous loci and they may have yielded mating-type locus structures in which only one of the idiomorphs is bordered by *APN2* and *SLA2*, and the other one is being located in a small independent contig. The two possibilities could in principle be differentiated by analyzing the fertility of single-spore isolates. Only PSN1167 permitted such analysis, as the other two strains were sterile. All the eight F1 single-spore isolates of PSN1167 were fertile, indicating that the strain was homothallic. Moreover, sequence reads across *APN2* (184 reads/kbp) and *SLA2* (195 reads/kbp) did not display higher coverage than those for *mat1-1-1* (196 reads/kbp), *mat1-1-2* (208 reads/kbp), *mat1-1-3* (224 reads/kbp) and *mat1-2-1* (206 reads/kpb), indicating that all six genes were present in the same number of copies, as expected if PSN1167 was homothallic. Indeed, one would expect heterothallics *APN2* and *SLA2* to have a higher coverage of sequence reads than the mating-type genes, i.e., to have the coverage of the *mat1-1* idiomorph added to the coverage of the *mat1-2* idiomorph. Coverage calculation for the two sterile strains, PSN850 and CBS 365.69, also suggested that they could be homothallics, although we could not test this directly. Indeed, the coverage calculations for PSN850 gave 202 reads/kbp for *APN2*, 226 reads/kbp for *SLA2*, 246 reads/kbp for *mat1-1-1*, 242 reads/kbp for *mat1-1-2*, 217 reads/kbp for *mat1-1-3*, reads/kbp for 238 *mat1-2-1*, and for CBS 365.69 1241 reads/kbp for *APN2*, 1090 reads/kbp for *SLA2*, 1289 reads/kbp for *mat1-1-1*, 1289 reads/kbp for *mat1-1-2*, 1216 reads/kbp for *mat1-1-3*, and 1019 reads/kbp for *mat1-2-1.*

Finally, six strains (PSN1062, PSN1175, PSN1104, PSTH200, PSN640 and PSTH195) had a single idiomorph, with either *mat1-1* or *mat1-2* bordered by *APN2* and *SLA2*. Such structures could be due to heterothallism with loss of one mating type in the sequenced isolate. It could also correspond to a particular form of homothallism, as some rare fungal species can undergo single-mating-type mating [44]. This could only be tested for the fertile PSN1062 and PSN1104. Mycelia from all F1 single spores isolated from PSN1062 (*n* = 5) produced mature-looking perithecia devoid of ascospores, showing that this strain underwent partial sexual reproduction with only one mating type, leaving open the question whether PSN1062 is homothallic or heterothallic. On the contrary, all mycelia from all single spore isolates from PSN1104 (*n* = 8) were sterile, albeit five out of eight differentiated small and barren perithecia. This showed that PSN1104 was heterothallic. The lack of the *mat1-1* mating type in the sequence assembly of PSN1062 and PSN1104 can be accounted for by a biassed ratio of nuclei, with the *mat1-1* muclei being in too low amounts to contribute significantly to the extracted DNA, while even a minute amount of *mat1-1* nuclei can permit mating. For the remaining four strains, the best explanation for their sterility is that these strains are heterothallic and that the cultures we have obtained have lost one mating type.

### 3.4. Taxonomy

#### 3.4.1. New Species

**Figure 13:**
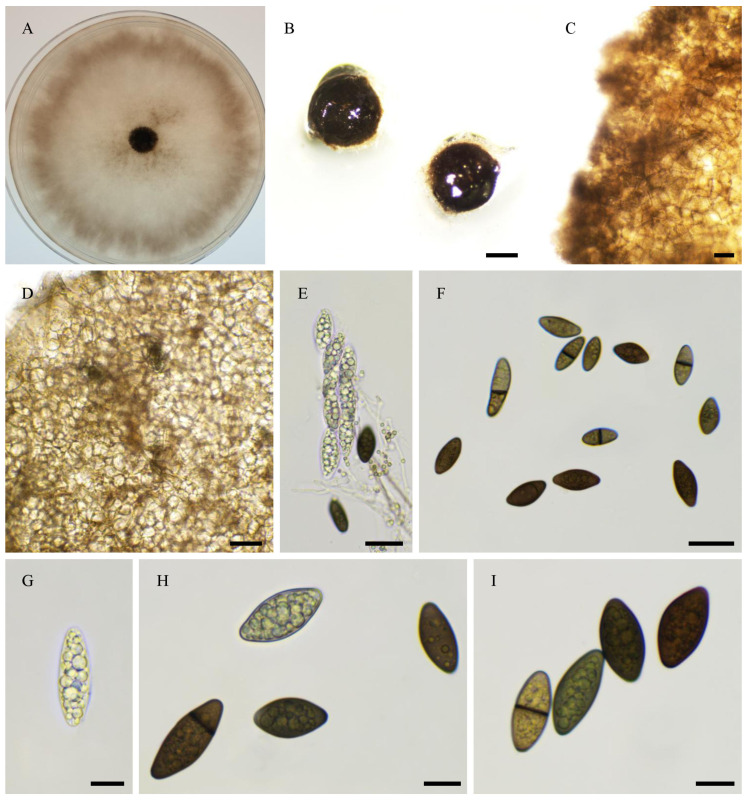
Morphology of *Pseudorhypophila gallica* sp. nov. PSN540. Mycelium on M2 medium after two weeks of growth (**A**). Perithecia (**B**). Perithecium wall is *textura angularis* (**C**,**D**). Asci and ascospores (**G**,**H**). Asci (**E**) and ascospores (**F**–**I**). Scale bars: **B** = 400 µm; **C**–**E** = 20 µm; **G**–**I** = 10 µm.

***Pseudorhypophila gallica***, Tangthirasunun and Silar **sp. nov.** Figure 13

Index fungorum number: 904,695.

Etymology: From the location of first two isolations.

Holotype: PC0820273; ex-type: PSN540 isolated from soil collected under bamboo in the public garden of Albert (Somme, Picardie), France in 2020; available under accession n° CIRM-BRFM 3856 at the “Centre International de Ressources Microbiennes—Champignons Filamenteux” (CIRM-CF, INRAE, France). Whole genome sequence available in GenBank under bioproject PRJNA1081049 for genomic data and PRJNA1339018 for transcriptomic data.

Other isolates: PSN2022 from St Maur sur le Loir (Eure et Loir), France–this strain did not reproducibly produce fruiting bodiest in vitro.

Description: Perithecia were readily differentiated on M0 + miscanthus after 2–3 weeks incubation at 27 °C with constant illumination; non-ostiolate, spherical, superficial or semi-immersed, and 761 ± 116 µm diam (*n* = 10). Their peridium was pale brown and semi-translucid, and tissue type was *textura angularis*. Paraphyses absent. Asci were eight-spored, clavate with a tapering and narrow apex. Ascospores were biseriate and bicellular. Ascospore heads were 25.4 ± 2.5 × 11.8 ± 1.6 μm ((21.5–)30.7 × 9.2(–17.1) μm, *n* = 30), ellipsoidal to oval, and hyaline at first, then green and brown and dark brown when matured. Few ascospore with 1-septate (slightly curved or straight) toward the apical, first median cell from base 14.8 ± 2.5 µm and the second median cell 10.0 ± 2.0 μm (*n* = 15).

Habitat and Distribution: Soil; this species has been found twice in two different regions of France, namely Picardie and Centre-Val de Loire.

Breeding system: Homothallic

In the ITS + LSU tree, the closest relative of *P. gallica* is *A. oryzae* CBS 376.74 and PSN2009 (Figure 1). In the phylogenomic tree, the closest relative is *P. oryzae* PSN2009 (Figure 12). They differ by the fact that the ascospores are at first biseriate in *P. gallica* and uniseriate in *P. oryzae*. Also, the ascospores of *P. gallica* are more elongated than those of *P. oryzae*: 25.4 ± 2.5 × 11.8 ± 1.6 μm *versus* 21.7 ± 2.2 × 13.2 ± 1.1 μm, respectively.

**Figure 14:**
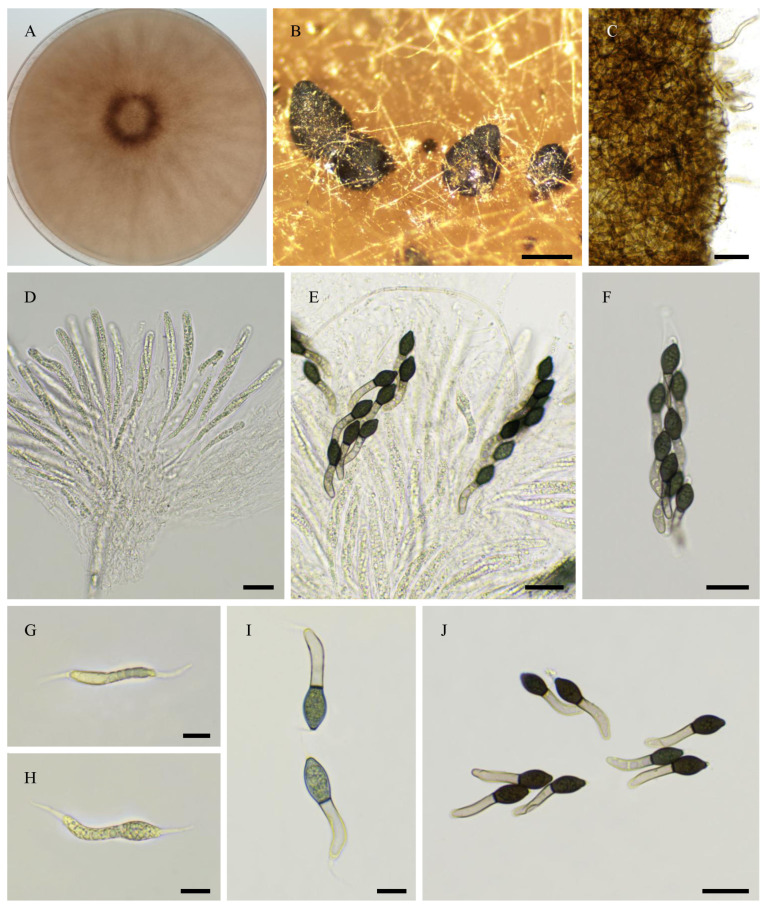
Morphology of *Pseudorhypophila guyanensis* sp. nov. PSN2406. Mycelium on M2 medium after two weeks of growth (**A**). Perithecia (**B**). Peridium with *textura angularis* (**C**). Young asci (**D**). Mature asci with ascospores (**E**,**F**). Young ascospores (**G**–**I**). Mature ascospores (**J**). Scale bars: **B** = 200 µm; **C**–**F**,**J** = 20 µm; **G**,**I** = 10 µm.

***Pseudorhypophila guyanensis***, Tangthirasunun and Silar **sp. nov.** (Figure 14).

Index Fungorum number: 904,696.

Etymology: From the location of first isolation.

Holotype: PC0820276; ex-type: PSN2406 isolated from soil taken in 2024 under a tree in a disturbed rainforest in French Guyana; available under accession n° CIRM-BRFM 3849 at the “Centre International de Ressources Microbiennes—Champignons Filamenteux” (CIRM-CF, INRAE, France). Whole genome sequence available in GenBank under Bioproject PRJNA1345941.

Description: Perithecia were readily differentiated on M0 + miscanthus after 2–3 weeks incubation at 27 °C with constant illumination; ostiolate, pyriform, superficial, or semi-immersed, 499 ± 66 µm diam (*n* = 10); their neck was black and slightly elongated. Their peridium was pale brown and semi-translucid, tissue type was *textura angularis*. Paraphyses absent. Asci were eight-spored, clavate with a tapering and narrow apex. Ascospores were biseriate and bicellular. They were at first vermiform, then the head enlarged and pigmented to yield *Cercophora*-like ascospores. Ascospore heads were 17.0 ± 1.0 × 9.6 ± 1.2 µm ((15.0–)18.6 × 6.4(–11.1) µm, *n* = 30), ellipsoidal with a slightly flattened base, and hyaline at first, then green and black when mature. The primary appendage was very long (often longer than the spore head,), flat, often curved, frequently collapsing, and 23.4 ± 2.2 × 5.6 ± 0.9 µm ((18.0–)25.5 × 4.6(–7.2) µm, *n* = 10). Secondary appendages were present at the apex of the spore head as a one-whip structure and at the end of the primary appendage as a whip.

Habitat and Distribution: Soil; this species has been found once from French Guyana.

Breeding system: Homothallic

*P. guyanensis* can be easily differentiated from the other *Pseudorhypophila* spp. by the fact that it differentiates ostiolate perithecia, like *P. mangenotii*. However, unlike *P. mangenotii*, which differentiate triangular ascospores with a rounded primary appendage, *P. guyanensis* differentiates *Cercophora*-like ascospores.

**Figure 15:**
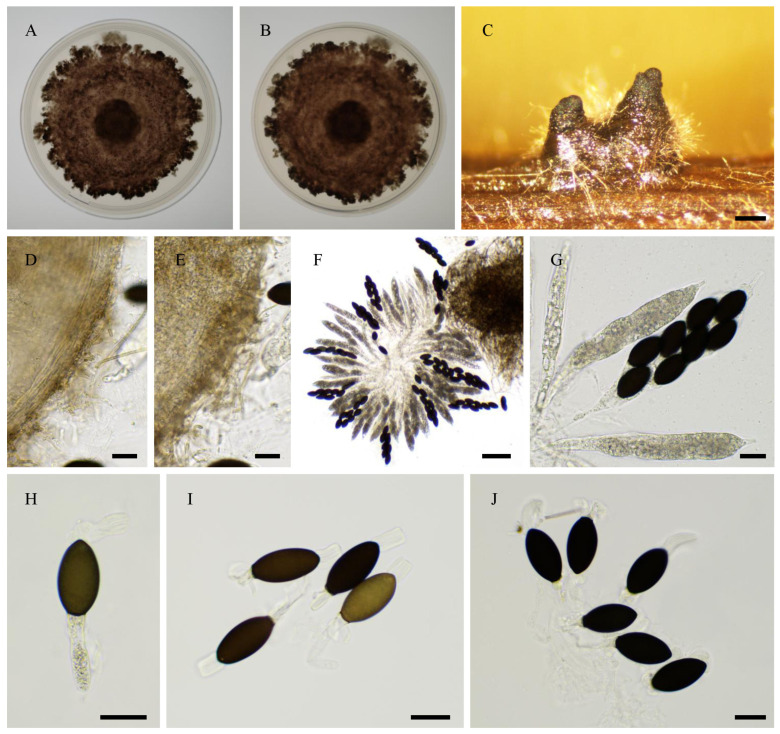
Morphology of *Rhypophila alpibus* sp. nov. PSN2022. Mycelium on M2 medium after two weeks of growth (**A**,**B**). Perithecia (**C**,**D**). Perithecia wall of *textura angularis* (**D**,**E**). Asci and ascospores (**F**,**G**), ascospores (**H**–**J**). Scale bars: **C** = 200 µm; **F** = 100 µm; **D**,**E**,**G**–**J** = 20 µm.

***Rhypophila alpibus***, Tangthirasunun and Silar **sp. nov.** Figure 15

Index Fungorum number: 904,697.

Etymology: From the location of first isolation.

Holotype: PC0820272; ex-type: PSN2212 isolated from cow dung collected in a mountain pasture in the Alps of Italy in 2024; available under accession n° CIRM-BRFM 3847 at the “Centre International de Ressources Microbiennes—Champignons Filamenteux” (CIRM-CF, INRAE, Marseille, France). Whole genome sequence available in GenBank under bioproject PRJNA1345941.

Description: Perithecia were readily differentiated on M0 + miscanthus after 2–3 weeks incubation at 27 °C with constant illumination; they were ostiolate, pyriform, superficial or semi-immersed, covered with numerous hairs, and 592 ± 100 µm diam (*n* = 10); their neck was black and slightly elongated. Their peridium was pale brown and semi-translucid, and tissue type was *textura angularis*. Paraphyses absent. Asci were eight-spored, clavate with a tapering and narrow apex. Ascospores were biseriate and bicellular. Ascospore heads were 42.9 ± 2.2 × 21.8 ± 1.1 µm ((39.0–)47.1 × 19.5(–24.2) µm, *n* = 30), ellipsoidal with a slightly flattened base, and hyaline at first, then green and black when matured. The primary appendage was very long (often longer than the spore head) and flat, frequently collapsing, and 50.5 ± 9.8 × 7.5 ± 1.8 µm ((36.4–)64.2 × 5.3(–10.8) µm, *n* = 7). Secondary appendages were present at the apex of the spore head as a fan-shaped structure of juxtaposed fibrils and at the junction between the spore head and the primary appendage as fibrils often two in number.

Habitat and Distribution: Dung; this species has been collected only once in the Alps mountains.

Breeding system: homothallic

Both in the ITS + LSU and phylogenomic tree, the closest relatives of *R. alpibus* are *R. camarguensis*, *R. decipiens*, *R. pleiospora* and *R. myriaspora*. Like *R. decipiens* and *R. camarguensis*, *R. alpibus* differentiated eight-spored asci, while *R. pleiospora* and *R. myriaspora* differentiated 16-spored and 64-spored asci, respectively. The ascospore heads of *R. alpibus* (42.9 ± 2.2 × 21.8 ± 1.1 µm) are larger than those of *R. decipiens* (35.5 ± 2.8 × 18.6 ± 1.2 µm) and *R. camarguensis* (36.0 ± 2.9 × 18.9 ± 1.3 µm).

**Figure 16:**
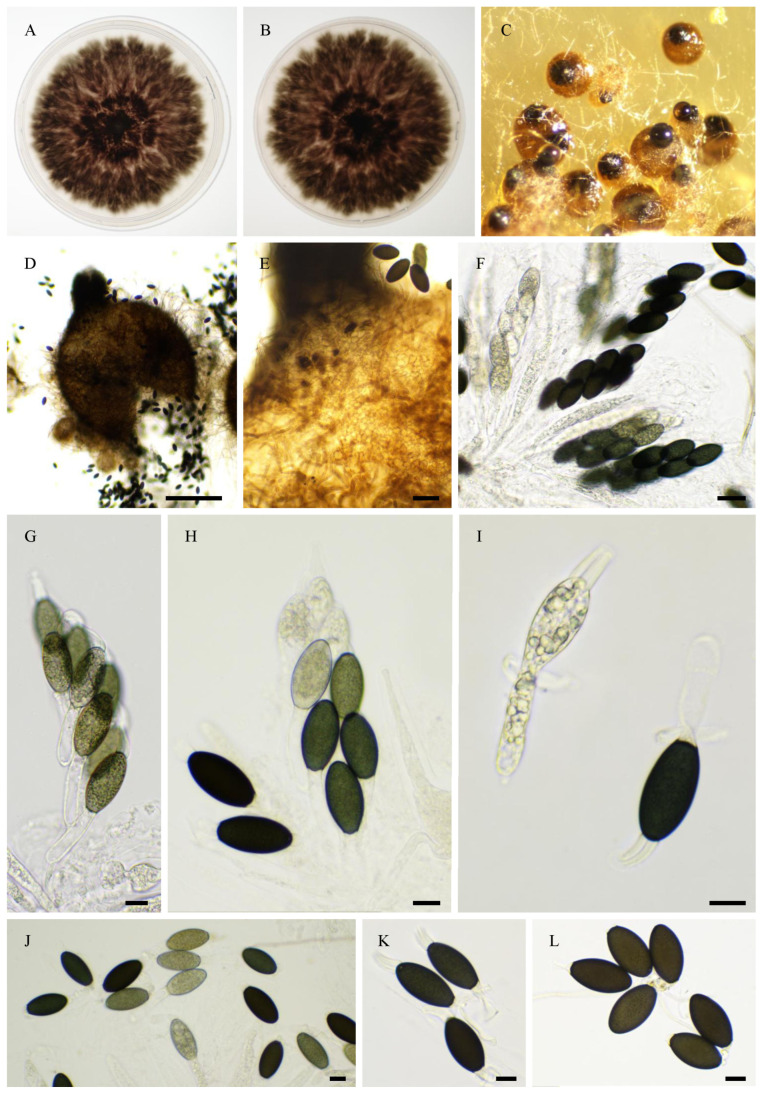
Morphology of *Rhypophila brasiliensis* sp. nov. PSN1104. Mycelium on M2 medium after two weeks of growth (**A**,**B**). Perithecia (**C**,**D**). Perithecia wall was with *textura angularis*. (**E**), Asci and ascospores (**F**–**H**). Ascospores (**I**–**L**). Scale bars: **D** = 200 µm; **E**,**F** = 20 µm; **G**–**L** = 10 µm.

***Rhypophila brasiliensis***, Tangthirasunun and Silar **sp. nov.** (Figure 16).

Index Fungorum number: 904,698.

Etymology: From the location of first isolation.

Holotype: PC0820275; ex-type: PSN1104 isolated from capybara dung collected in the Parc Taquaral, Campinas, Brazil in 2021; available under accession n° BRFM 3859 at the “Centre International de Ressources Microbiennes—Champignons Filamenteux” (CIRM-CF, INRAE, Marseille France). Whole genome sequence available in GenBank under bioproject PRJNA1345941.

Description: Perithecia were readily differentiated on M0 + miscanthus after 2–3 weeks incubation at 27 °C with constant illumination; they were ostiolate, pyriform, superficial or semi-immersed, and 498 ± 44 µm diam (*n* = 10); their neck was black and slightly elongated. Their peridium was pale brown and semi-translucid, and tissue type was *textura angularis*. Paraphyses absent. Asci were eight-spored, clavate with a tapering and narrow apex. Ascospores were biseriate and bicellular. Ascospore heads were 33.2 ± 2.0 × 17.2 ± 1.6 µm ((28.1–)38.4 × 14.8(–22.5) µm, *n* = 30), ellipsoidal with a slightly flattened base, and hyaline at first, then green and black when matured. The primary appendage was very long (often longer than the spore head) and flat, frequently collapsing, and 34.6 ± 4.1 × 7.3 ± 0.7 µm ((28.7–)41.1 × 5.9(–8.1) µm, *n* = 10), Secondary appendages were present at the apex of the spore head as a whip-shaped or fibrillate or lamellate structure. No obvious appendage(s) is seen at the base of the spore head.

Habitat and Distribution: Dung, isolated once in Brazil.

Breeding system: Heterothallic

In the ITS + LSU, the closest unequivocal relative of *R. brasiliensis* was *R. cochleariformis*. They differ by the number of ascospores per ascus (eight for *R. brasiliensis* and 128 for *R. cochleariformis*; [46]). In the phylogenomic tree, *R. brasiliensis* is related to *R. reunionensis* and *R. thailandica*. *R. reunionsensis* produced 64-spored asci and *R. thailandica* eight-spored asci. However, the ascospore heads of *R. brasiliensis* are more elongated than those of *R. thailandica*: 33.2 ± 2.0 × 17.2 ± 1.6 µm *versus* 31.9 ± 3.3 × 19.8 ± 1.7 µm.

**Figure 17:**
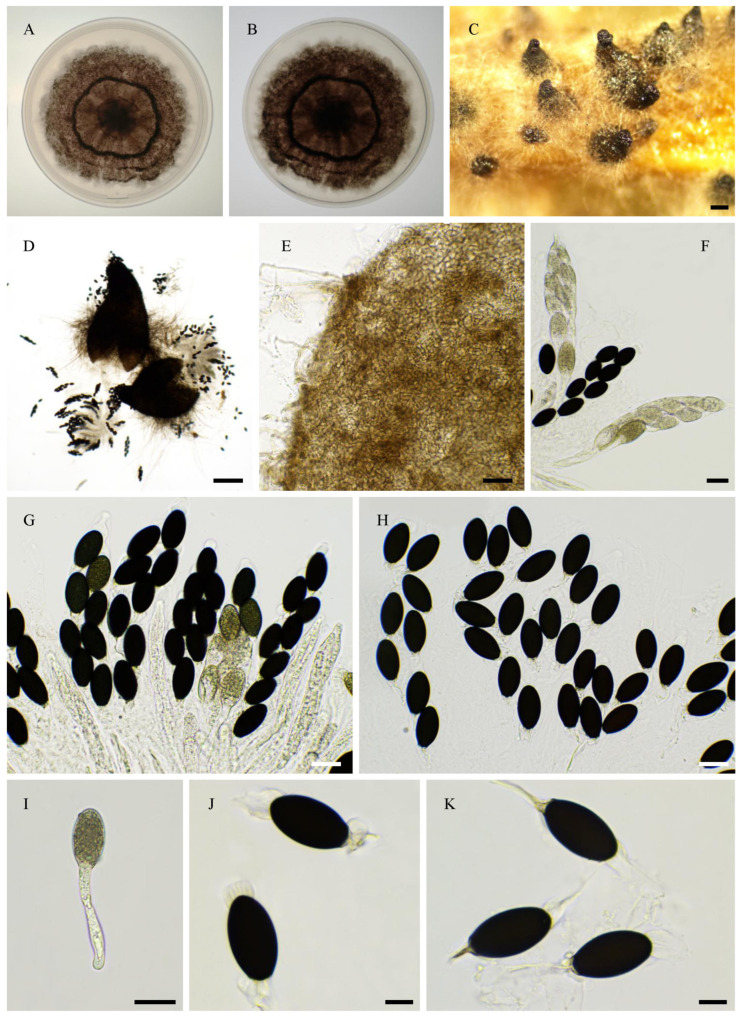
Morphology of *Rhypophila camarguensis* sp. nov. PSN673. Mycelium on M2 medium after two weeks of growth (**A**,**B**). Perithecia (**C**,**D**). Perithecium wall with *textura angularis* (**E**). Asci and ascospores (**F**,**G**). Mature ascospores (**H**,**J**,**K**). Young ascospore (**I**). Scale Bars: **C**,**D** = 200 µm; **E**–**I** = 20 µm; **J**,**K** = 10 µm.

***Rhypophila camarguensis***, Tangthirasunun and Silar **sp. nov.** Figure 17

Index Fungorum number: 904,699.

Etymology: From the location of first isolation.

Holotype: PC0820277; ex-type: PSN673 isolated from cow dung collected in 2021 in Camargue, France; available under accession n° CIRM-BRFM 3845 at the “Centre International de Ressources Microbiennes—Champignons Filamenteux” (CIRM-CF, INRAE, France). Whole genome sequence available in GenBank under bioproject PRJNA1345941.

Description: Perithecia were readily differentiated on M0 + miscanthus after 2–3 weeks incubation at 27 °C with constant illumination; they were ostiolate, pyriform, superficial or semi-immersed, covered with numerous hairs, and 510 ± 51 µm diam (*n* = 10); their neck was black and slightly elongated. Their peridium was pale brown and semi-translucid, tissue type was *textura angularis*. Paraphyses absent. Asci were eight-spored, clavate with a tapering and narrow apex. Ascospores were biseriate and bicellular. Ascospore heads were 36.0 ± 2.9 × 18.9 ± 1.3 µm ((27.8–)39.7 × 16.5(–21.0) µm, *n* = 30), and ellipsoidal with a slightly flattened base, and hyaline at first, then green and black when matured. The primary appendage was very long (often longer than the spore head) and flat, frequently collapsing, and 54.0 ± 4.6 × 7.4 ± 0.8 ((46.0–)58.9 × 5.9(–8.5) µm, *n* = 10). Secondary appendages were present at the apex of the spore head as a fan-shaped structure of juxtaposed fibrils and at the junction between the spore head and the primary appendage as fibrils often two in number.

Habitat and Distribution: Dung; collected once in the South of France

Breeding system: Homothallic

In the ITS + LSU and phylogenomic trees, *R. camarguensis* is closely related to the other eight-spored species of *Rhypophila*, *R. decipiens* and *R. alpibus*. Its ascospore heads are smaller than those of *R. alpibus* (36.0 ± 2.9 × 18.9 ± 1.3 µm *versus* 42.9 ± 2.2 × 21.8 ± 1.1 µm) and are similar to those of *R. decipiens*, which are 35.5 ± 2.8 × 18.6 ± 1.2 µm. *R. decipiens* and *R. camarguensis* can easily be differentiated by their mycelium, which is darker on M2 medium for *R. camarguensis* and by their genome sequence and their ITSs differ by 15 differences.

**Figure 18:**
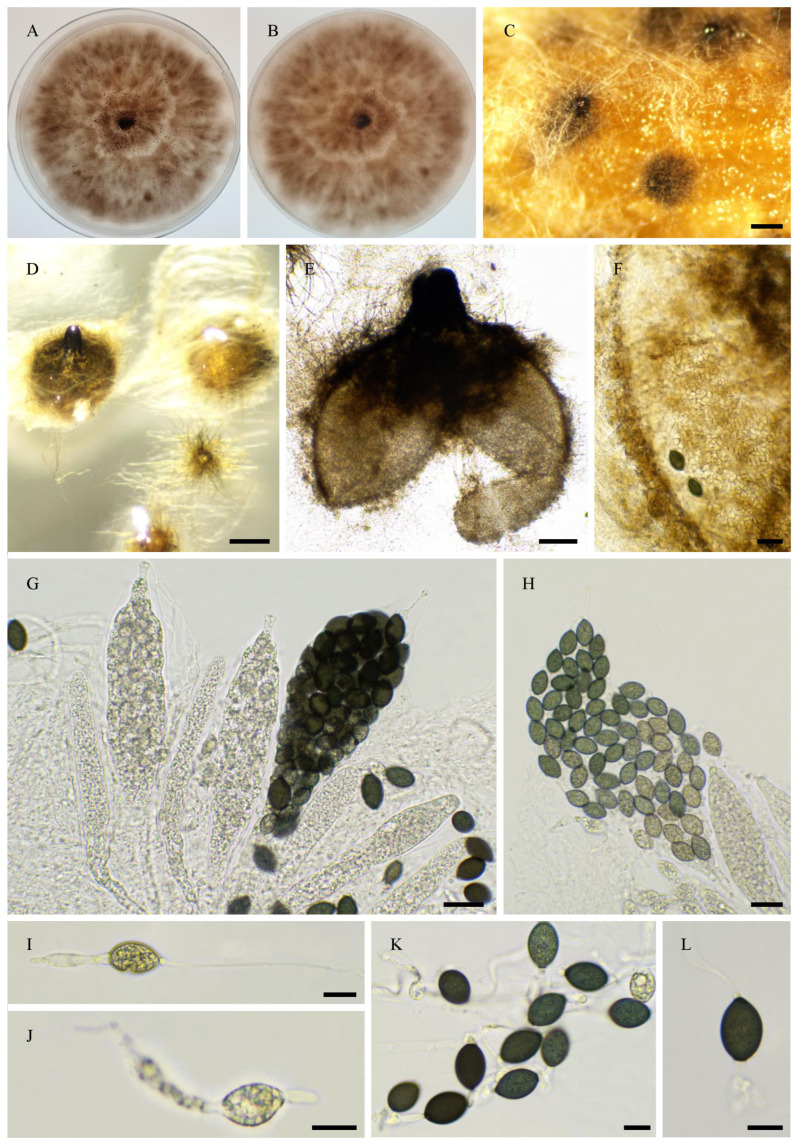
Morphology of *Rhypophila reunionensis* sp. nov. PSN1167. Mycelium on M2 medium after two weeks of growth (**A**,**B**). Perithecia (**C**,**D**). Perithecia with neck (**E**). Peridium with *textura angularis* (**F**). Asci with ascospores (**G**,**H**). Yong ascospores (**I**,**J**). Mature ascospores (**K**,**L**). Scale bars: **C**,**D** = 200 µm; **E** = 100 µm; **F**–**H**= 20 µm; **I**–**L** = 10 µm.

***Rhypophila reunionensis***, Tangthirasunun and Silar **sp. nov.** Figure 18

Index Fungorum number: 904,700.

Etymology: From the location of first isolation.

Holotype: PC0820274; ex-type: PSN1167 isolated from cow dung collected near Piton de la Fournaise in Ile de la Réunion, oversea France in the Indian Ocean; available under accession n° CIRM-BRFM 3844 at the “Centre International de Ressources Microbiennes—Champignons Filamenteux” (CIRM-CF, INRAE, Marseille France). Whole genome sequence available in GenBank under bioproject PRJNA1345941.

Description: Perithecia were readily differentiated on M0 + miscanthus after 2–3 weeks incubation at 27 °C with constant illumination; There were covered with hairs, ostiolate, pyriform, superficial or semi-immersed, and 558 ± 63 µm diam (*n* = 10); their neck was black and slightly elongated. Their peridium was pale brown and semi-translucid, and tissue type was *textura angularis*. Paraphyses absent. Asci were 64-spored, clavate with a tapering and narrow apex. Ascospores were irregularly arranged., Ascospore heads were 19.6 ± 1.3 × 12.8 ± 0.8 µm ((16.9–)22.1 × 11.5(–14.6) µm, *n* = 30), ellipsoidal with a slightly flattened base, and hyaline at first, then green and black when matured. The primary appendage was very long (often longer than the spore head) and flat, frequently collapsing, and 30.8 ± 5.7 × 4.9 ± 1.0 µm ((19.5–)40.6 × 2.9(–6.1) µm, *n* = 10), Secondary appendages were present at the apex of the spore head as a rot-shaped, apical gelatinous caudae absent or present.

Habitat and Distribution: Dung; collected only once in Ile de la Réunion in the Indian Ocean.

Breeding system: Homothallic

*R. reunionensis* is related to *R. myriaspora*, the other 64-spored species known in the *Rhypophila* genus. It has smaller ascospore heads than *R. myriaspora*: 19.6 ± 1.3 × 12.8 ± 0.8 µm *versus* 27.3 ± 2.8 × 17.1 ± 1.2 µm.

**Figure 19:**
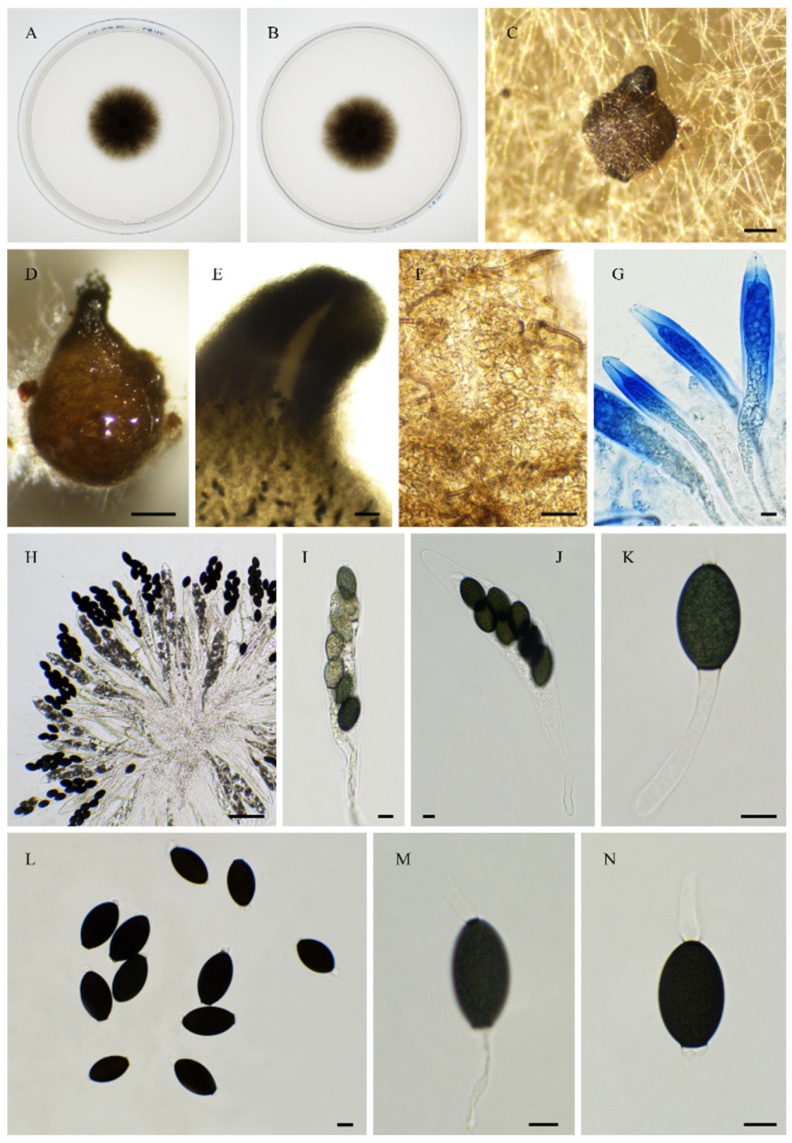
Morphology of *Rhypophila thailandica* sp. nov. PSTH81. Mycelium on M2 medium at 6 days (**A**,**B**). Perithecia (**C**,**D**). Neck of perithecia with visible ostiole (**E**). Peridium with *textura angularis* (**F**). Young asci (**G**). Asci and ascospores (**H**–**J**). Ascospores (**K**–**N**). Scale bars: **C**,**D** = 200 µm; **E**,**G** = 100 µm; **F** = 20 µm; **H**–**N** = 10 µm.

***Rhypophila thailandica***, Tangthirasunun and Silar **sp. nov.** (Figure 19).

Index Fungorum number: 904,701.

Etymology: from the location of first isolation

Holotype: PC0820271; ex-type: PSTH81 isolated from elephant dung collected in Ayuthaya, Thailand, in 2023; available under accession n° CIRM-BRFM 3843 at the “Centre International de Ressources Microbiennes—Champignons Filamenteux” (CIRM-CF, INRAE, Marseille, France). Whole genome sequence available in GenBank under bioproject PRJNA1345941.

Description: Perithecia were readily differentiated on M0 + miscanthus after 2–3 weeks incubation at 27 °C with constant illumination; they were ostiolate, pyriform and 527 ± 103 µm diam (*n* = 10); their neck was black and slightly elongated. Their peridium was pale brown and semi-translucid, and tissue type was *textura angularis*. Paraphyses were absent. Asci were eight-spored, clavate with a tapering and narrow apex. Ascospores were biseriate and bicellular. Ascospore heads were 31.9 ± 3.3 × 19.8 ± 1.7 µm ((25.2(–41.9) × 16.1(–22.9) µm, *n* = 30), ellipsoidal with a slightly flattened base, and hyaline at first, then green and black when matured. The primary appendage was very long (often longer than the spore head) and flat, frequently collapsing, and 49.3 ± 3.3 × 7.2 ± 1.5 µm ((42.6(–54.0) × 5.4–10.3) µm, *n* = 10). Secondary appendages were present at the apex of the spore head as a whip-shaped or fibrillate or lamellate structure.

Habitat and Distribution: Dung, isolated once in Thailand.

Breeding system: Homothallic

In the ITS + LSU tree, the closest relative of *R. thailandica* is *R. cochleariformis* (Figure 1). In the phylogenomic tree, the closest relative is *R. reunionensis* (Figure 12). The three species differ by the number of ascospores per ascus, with eight for *R. thailandica*, 64 for *R. reunionensis* and 128 for *R. cochleariformis*.

#### 3.4.2. New Combinations

***Pseudorhypophila latipes*** (N. Lundq., ex Malloch and Cain) Tangthirasunun and Silar **comb. nov.** IF = 904,702 (Figure 8).

Basionym: *Tripterospora latipes* N. Lundq., IF = 340,467

Synonym: *Zopfiella latipes* (N. Lundq.) Malloch and Cain, IF = 325,683

Material examined: Living cultures of IMI350600 and PSQ110, whose genome sequences are available in GenBank under bioproject PRJNA1345941.

Morphological analysis of both IMI350600 and PSQ110 is consistent with the original description of *Tripterospora latipes* Lundq. IMI350600 is the ex-type of the species. Phylogenetic analysis based on 2798 genes clearly places both strains in the *Pseudorhypophila* genus. This species is homothallic.

***Pseudorhypophila oryzae*** (Carolis and Arx) Tangthirasunun and Silar **comb. nov.** IF = 904,703 (Figure 9).

Basionym: *Apodus oryzae* Carolis and Arx, IF = 308,867.

Material examined: Living cultures of PSN2009, whose genome sequence is available in GenBank under bioproject PRJNA1345941.

Morphological analysis of PSN2009 is consistent with the original description of *A. oryzae* Carolis and Arx. PSN2009 has no difference with *A. oryzae* CBS 376.74 in its ITS and LSU, and a single difference in TUB2, placing both strains in the same species. Phylogenetic analysis based on 2798 genes clearly places PSN2009 in the *Pseudorhypophila* genus. This species is homothallic.

## 4. Discussion

*Sordariales* is an important order of fungi [17]. It hosts species-producing enzymes and secondary metabolites that are of interest for industrial purposes [17], including some made by several thermophilic fungi [47,48,49]. It also hosts three important models used in genetical and biochemical studies, namely *N. crassa*, *Sordaria macrospora* and *P. anserina*, putting them among the best-known fungi [17]. Yet, although species of *Sordariales* are among the most frequent species present in soils [15] and dung [16], the overall diversity of the order is poorly known. In particular, several well-studied species were found to be complexes of cryptic species [4,5,6,7,8,9,10]. Here, we tackle a combination of genome sequences and morphological analyses of the diversity of *Naviculisporaceae*, which is one of the families hosted by *Sordariales*.

Firstly, by comparing the DNA barcodes (ITS, LSU, *TUB2*, *RPB2*, *gpd*…) extracted from our genome sequences to that of previously characterized strains, we found that many isolates attributed to the same species presented high levels of nucleotide differences. This might result from sequencing errors or be the results of misidentifications in the collections, as previously exemplified [4]. However, sequence differences could also be indicative of the presence of complexes of cryptic species. The availability of only limited barcode sequences is not enough to disentangle the different possibilities, especially when the isolates do not fruit or no living culture is available, so that the morphology of the strain cannot be compared with the original description. In the case of *Sordariales*, the task is rendered more complex by the fact that some species, which were described a long time ago, have missing holotypes, have insufficient description in the protologue or holotypes of historical values, or are in so poor condition that they cannot be used to extract DNA. Here, for example, we suggest that strain IFO 9826 may not belong to *P. latipes* and that the strains of the previously analyzed *P. araneosa* (F-116,361, TNM F17207 and ATCC 36,386) and our newly isolated strain PSN1062 may belong to different species. Availability of complete genome sequence can on the contrary settle some issues. Indeed, comparison using FungANI [2] of the genome sequence of CBS 256.69 (labelled as *R. decipiens* in the collection) to that of PSN2105, for which morphology has been determined, shows that both belong to the same species, *R. myriaspora*. The diminishing cost of next-generation sequencing and the ease with which raw data can be assembled using Unicycler that freely runs on Galaxy platforms make it possible for any mycologist with a limited knowledge of bioinformatics to obtain genome sequence useful for species delimitation. The determination of the barcodes that are presently recommended for fungal identification [50] is often time-consuming, as it often requires several PCR amplifications in different conditions to obtain a significant set of barcodes (nowadays, studies often determine four barcodes: ITS, LSU, *TUB2* and *RPB2*). Then, Sanger sequencing must be performed. While the Sanger sequencing can be externalized, PCR amplifications usually cannot. Overall, with slightly higher cost and less intensive internal work, genome sequences can be used for species delimitation with little efforts and provide a wealth of additional data, including, for example, the possibility to determine the structure of the mating-type loci. We therefore advocate that complete genome sequencing becomes the gold standard for the description of the new species.

Analyses of the mating-type loci showed that most strains had mating-type loci similar to homothallic *Sordariales* such as *S. macrospora* [43], with the *mat1-1* and *mat1-2* idiomorphs located at the same position in the genome between the *APN2* and *SLA2* genes. However, some homothallic *Naviculisporaceae* had atypical *mat* loci with the two haplotypes at two different locations in the genome. This situation is not unprecedented and has also been observed in homothallic *Chaetomiaceae*, another *Sordariales* family [51]. Additionally, one *Naviculisporaceae* strain underwent partial sexual reproduction, i.e., the making of mature-looking barren perithecia, with a single mating type. Among the ten strains that did not fructify in vitro, four seemed to carry only one of the two idiomorphs of heterothallics, indicating that their sterility in vitro might primarily be due to the lack of an appropriate mate to initiate sexual reproduction. However, the other sterile strains appeared to carry the whole complement of *mat* genes (i.e., *mat1-1-1*, *mat1-1-2*, *mat1-1-3* and *mat1-2-1*), suggesting that they required special conditions to fructify that we were not able to provide. These may include the presence of special metabolite(s) or microorganism(s) present in their natural environment.

In addition to the genomes for the ex-types of the new species described here, our study provides genome references for well-known species such as *R. myriaspora* and *P. mangenotii*, which are the type species of *Rhypophila* and *Pseudorhypophila*, respectively. Phylogenomic analysis clearly confirms the separation of the two genera. It also suggests that additional data (i.e., the determination of the genomes and morphology of additional species) are required for *Gimaniella* to be definitively validated; so far, the two strains known for this genus do not undergo sexual reproduction in vitro. Additional strains are also needed to obtain a better idea of the diversity of the genus *Naviculispora* and to obtain some sequences for the genus *Areotheca* that we have not encountered during our strain isolation.

As shown in Figure 1, many species lack an identified type for which sequences are available. Many of these were described a long time ago. Their original description might be too scant to accurately identify them and/or a type could be missing, the latter could be of historical value or could be in too a poor condition to make a reliable identification (see, for example, refs [16,17]). Here, we introduce four eight-spored *Rhypophila* species that closely resemble *R. decipiens*: *R. alpibus*, *R. brasiliensis*, *R. camarguensis* and *R. thailandica*. All five species differentiate very similar fruiting bodies and asci-containing biseriate ascospores having a very long primary appendage. They appear to differ by the sizes of the ascospore heads, shape and presence of the secondary appendages, and/or mycelium morphology. Similarly, we introduce *R. reunionsensis*, another 64-spored species closely resembling *R. myriaspora*, but with smaller ascospore heads. We also introduce *P. gallica*, an eight-spored species closely resembling *P. oryzae*, having more elongated ascospores. All these species can be clearly differentiated by their genome sequences. The phylogenomic tree shows that the sixty-four-spored species, along with the sixteen-spored *R. pleiospora*, are intermingled with the eight-spored ones, indicating recurrent changes in the number of ascospores per ascus. Because a single strain for most species has been analyzed, we do not know the intraspecific variability of the size of the ascospores, which in other *Sordariales* species can be substantial [4,10]. More strains for each species must therefore be analyzed before fully accepting this character as discriminant. Moreover, the *Sordariales* species that have been thoroughly investigated turned out to actually be complexes of cryptic species, indistinguishable by looking at the morphology of the sexual reproductive structures [4,5,6,7,8,9,10]. So far, we have sequenced one or two strains for each *Naviculisporaceae* species, which is insufficient to detect complexes of closely related cryptic species. We thus advocate that before epitypification, one awaits the sequences of several additional strains. We also advocate that more than four DNA barcodes should be determined for the types of some species, such as *P. araneosa* or *A. japonense*, so as to unequivocally identify newly isolated strains, such as PSN1062 and PSN1175.

## 5. Conclusions

Through the isolation of new and collection strains, combined with genome sequencing and morphological analyses, our study improves the knowledge of the diversity of *Naviculisporaceae*. Genome sequences are provided for seven previously known species, some of which are commonly found on dung or in soil (e.g., *R. myriaspora* and *P. mangenotii*, the type species for the *Rhypophila* and *Pseudorhypophila* genera), in addition to that of the widespread and common *R. decipiens* that was previously published. Seven species new to science are described and eight strains, which may belong also to species new to science, await further characterization. A phylogenomic tree of the family with 100% statistical support for all nodes is provided.

## Figures and Tables

**Table 1:** Strain origins and main characteristics of their genomes. Taxonomic novelties are indicated in bold.

Strain	Species (Other Names *)	Isolation Year	Origin	Substrate	Sequencing Palteform	Genome Size (Mb)	Probable Breeding System
PSN1062	*Podospora aff. araneosa*	2021	Alps, France	Hare dung	Novogene	40.3	heterothallic
PSN850	*Naviculispora* sp.	2021	Camargue, France	Horse dung	Novogene	41.8	heterothallic
CBS 365.69	*Arnium leporinum* (*Podospora leporina*)	1969	The Netherlands	Rabbit dung	JGI	48.5	heterothallic
PSN1175	*Arnium aff. japonense*	2021	Kobarld, Slovenia	Deer dung	Biomics	49.6	heterothallic
PSTH81	***Rhypophila thailandica* sp. nov.**	2023	Ayuthaya, Thailand	Elephant dung	Novogene	50.3	homothallic
PSN1167	***Rhypophila reunionensis* sp. nov.**	2022	La Réunion Island, France	Cow dung	Novogene	46.3	heterothallic
PSN1104	***Rhypophila brasiliensis* sp. nov.**	2021	Campinas, Brazil	Capybara dung	Biomics	45.9	heterothallic
PSN637	*Rhypophila* sp.	2021	Ontario, Canada	White rabbit dung	JGI	46.9	homothallic
PSN673	***Rhypophila camarguensis* sp. nov.**	2021	Camargue, France	Cow dung	Novogene	45.9	homothallic
PSN293 ^$^	*Rhypophila decipiens* (*Podospora decipiens*)	2017	Auvergne, France	Donkey dung	JGI	47.1	homothallic
PSN2105	*Rhypophila myriaspora*	2024	Picardie, France	Horse dung	Novogene	48.2	homothallic
CBS 256.69	*Rhypophila myriaspora* (*Rhypophila decipiens*)	1969	The Netherlands	Rabbit dung	JGI	48.3	homothallic
PSN658	*Rhypophila pleiospora*	2021	Ontario, Canada	*Sylvillagus floridanus* dung	JGI	46.7	homothallic
PSN2212	***Rhypophila alpibus* sp. nov.**	2024	Alps, Italy	Cow dung	Novogene	47.9	homothallic
PSTH200	*Gilmaniella* sp.	2023	Chonburi, Thailand	Elephant dung	Novogene	51.8	heterothallic
PSN640	*Pseudorhypophila* sp.	2021	Chad	Camel dung	JGI	47.6	heterothallic
PSTH195	*Pseudorhypophila sp.*	2023	Chiang Rai, Thailand	Soil	Novogene	50.6	heterothallic
IMI229743	*Pseudorhypophila marina* (*Zopfiella marina*)	1978	Japan	Marine mud	JGI	53.7	homothallic
IMI350600	***Pseudorhypophila latipes *****comb. nov.** (*Zopfiella latipes*)	1991	U.K.	Not stated by the provider	Novogene	52.3	homothallic
PSQ110	***Pseudorhypophila latipes* comb. nov.**	2019	Québec, Canada	Soil	Novogene	52.6	homothallic
IMI229747	*Pseudorhypophila mangenotii* (*Triangularia mangenotii*)	1978	Japan	Soil	JGI	54.3	homothallic
PSN2406	***Pseudorhypophila guyanensis* sp. nov.**	2025	Guyane	Soil	Novogene	52.6	homothallic
PSN2407	*Pseudorhypophila* sp.	2025	Guyane	Soil	Novogene	52.8	homothallic
PSN2009	***Pseudorhypophila oryzae* comb. nov.**	2024	Centre-Val de Loire, France	Soil	Novogene	56.2	homothallic
PSN540	***Pseudorhypophila gallica* sp. nov.**	2020	Picardie, France	Soil	JGI	55.4	homothallic
PSN2022	***Pseudorhypophila gallica* sp. nov.**	2024	Centre-Val de Loire, France	Soil	Novogene	54.6	homothallic

*: names under which the strain is commercially available or was sequenced; $: PSN293 genome sequence was previously reported [14].

**Table 2:** ITS and LSU accession numbers of the strains used in the ITS + LSU phylogenetic analysis.

Strain	Taxa	ITS	LSU
CBS 118,394	*Apodospora peruviana*	EU573703	KF557665
CBS 506.70 ^T^	*Apodus deciduus*	NR_145141.1	NG_056953
CBS 376.74	*Apodus oryzae*	AY68120	AY681166
CBS 215.60	*Areotheca ambigua*	AY999137	AY999114
UAMH 7495	*Areotheca areolata*	AY587911	AY587936
Lundqvist 7098-e	*Arnium caballinum*	NA	KF557672
SANK 10,273	*Arnium japonense*	NA	KF557680
Lundqvist20874-c	*Arnium mendax*	NA	KF557687
E00122117	*Arnium mendax*	NA	KF557688
NBRC 9235 ^T^	*Gilmaniella humicola*	available at https://www.nite.go.jp/nbrc/catalogue/ (accessed on 11 October 2025)	
CBS 137,295 ^T^	*Naviculispora terrestris*	MT784136	KP981439
NTUPPMCC 22-297 ^T^	*Pseudorhypophila formosana*	PV476805	PV476867
CBS 155.77 ^T^	*Pseudorhypophila marina*	MK926851	MK926851
CBS 698.96	*Pseudorhypophila marina*	MK926853	MK926853
CBS 413.73 ^T^	*Pseudorhypophila pilifera*	MK926852	MK926852
CBS 419.67 ^T^	*Pseudorhypophila mangenotii*	MT784143	KP981444
CBS 249.71	*Rhypophila cochleariformis*	AY999123	AY999098
CBS 258.69	*Rhypophila decipiens*	KX171946	AY780073
TNM F17211	*Rhypophila myriaspora* ^$^	EF197083	NA
TNM F16889	*Rhypophila pleiospora*	EF197084	NA
F-116,361	*Sordaria araneosa*	FJ175160	NA
IFO9826	*Zopfiella latipes*	AY999129	AY999107

^T^: type; ^$^: Marin–Felix et al. [11] labelled this strain as *R. myriaspora* in their phylogenetic tree, which is the name classically used, and *R. myriospora* in their diagnosis, which is the name now retained in several databanks. In the present paper, we use the traditional name of *R. myriaspora*, as the protolog described the species as *Sordaria myriaspora* Crn. Mscr. [29]. NA: not available.

**Table 3:** Average nucleotide identity (ANI) between selected *Rhypophila* spp. strains. The ANI value in red identifies strains assigned to the same species.

	PSN2212	PSN2105	CBS 256.69	PSN293
**PSN658**	94.99%	95.07%	95.08%	95.17%
**PSN2212**		95.08%	95.09%	95.21%
**PSN2105**			99.74%	95.20%
**CBS 256.69**				95.20%

**Table 4:** Average nucleotide identity (ANI) between selected *Pseudorhypophila* spp. strains. The ANI value in red identifies strains assigned to the same species.

	PSQ110	IMI350600	IMI229747	PSN2406	IMI229743	PSN540	PSN2022	PSN2407	PSN2009
**PSTH195**	87.90%	88.47%	88.49%	87.75%	89.14%	87.86%	87.86%	87.85%	87.87%
**PSQ110**		99.26%	87.91%	87.25%	88.36%	87.34%	87.36%	87.28%	87.42%
**IMI350600**			87.93%	87.25%	88.37%	87.38%	87.38%	87.32%	87.46%
**IMI229747**				88.25%	89.02%	88.35%	88.36%	88.35%	88.35%
**PSN2406**					87.78%	94.18%	94.18	94.10%	94.00%
**IMI229743**						87.90%	87.91%	87.84%	87.95%
**PSN540**							98.69% *	96.28%	97.26%
**PSN2022**								96.43%	97.30%
**PSN2407**									96.44%

* Although ANI is lower than 99%, PSN540 and PSN2022 belong to the same species. Low ANI was the result of the presence of about 4% of DNA regions diverging by 10–15% (see Figure 7).

## Data Availability

All newly isolated strains can be purchased at CIRM-CF (https://www.cirm-fungi.fr/, accessed on 11 October 2025). NGS Raw data are available in the GenBank SRA repository (see Table S1 for accession numbers) and/or the JGI MycoCosm website (https://mycocosm.jgi.doe.gov, accessed on 11 October 2025). Assemblies can be downloaded from a GitHub repository (https://github.com/podo-gec/fungi-public-data/tree/master/naviculisporaceae, accessed on 11 October 2025) or the JGI MycoCosm website (https://mycocosm.jgi.doe.gov, accessed on 11 October 2025).

## Supplementary Materials

The following supporting information can be downloaded at https://www.mdpi.com/article/10.3390/jof11120880/s1, Figure S1: Morphology of *Pseudorhypophila latipes* PSQ110; Table S1: Main features of the genomes used for phylogenomic analysis.

## Abbreviations

The following abbreviations are used in this manuscript:

ANI	Average nucleotide identity
ITS	Internal transcribed spacer
kbp	Kilobase-pair
LSU	rDNA large subunit
RPB2	RNA polymerase II subunit 2
TUB2	β-tubulin
