# Supplementary material for: Diversity of Sordariales Fungi: Identification of Seven New Species of Naviculisporaceae Through Morphological Analyses and Genome Sequencing
Source: J Fungi (Basel). 2025 Dec 12;11(12):880. doi: 10.3390/jof11120880 (PMC12734299; doi:10.3390/jof11120880)
Supplement: Supplementary file 1 [file jof-11-00880-s001.zip › jof-3998686-Figure.pdf]

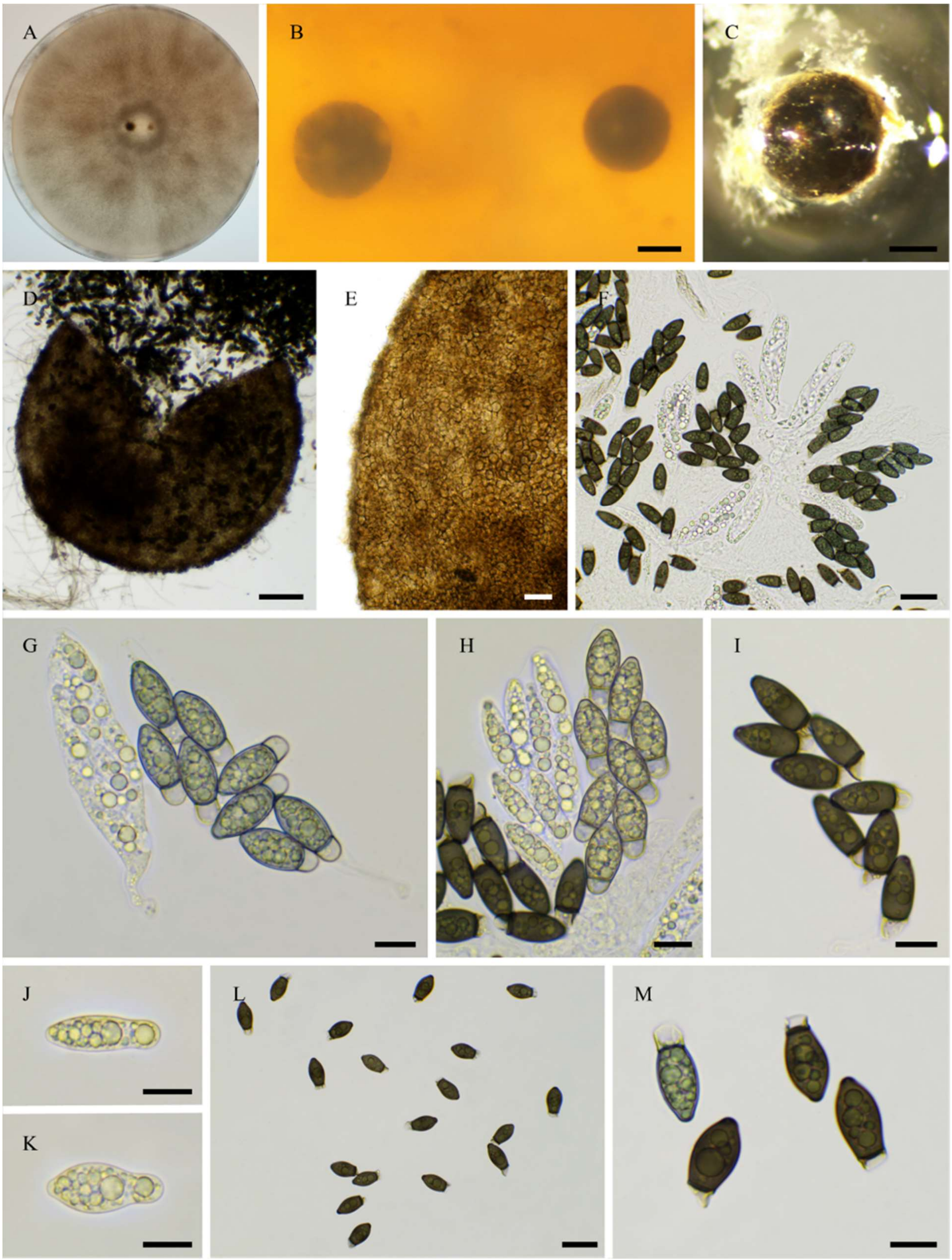

Figure S1. Morphology of *Pseudorhypophila latipes* PSQ110. Mycelium on M2 medium after two weeks of growth (A). Perithecia, non-ostiolate, were readily differentiated on M0 + miscanthus after 2-3 weeks incubation at 27°C with constant illumination; superficial or immersed, black, globose to subglobose, they were 522 ± 99 µm diam (n=10) (B-D). Their peridium was pale brown and semi-translucid, tissue type was *textura angularis* (E). Asci were 8-spored, clavate with a tapering and narrow apex (F-I). Ascospores were biseriate and bicellular (G-I). Ascospore heads were 16.9 ± 1.5 × 8.4 ± 0.9 µm, n=30), ovoid with a slightly flattened base, hyaline at first (J-K), then green (G, M) and brown to dark brown when mature (L-M). The primary appendage had a rounded base and hyaline (G), frequently collapsing (L-M), they were 5.6 ± 1.1 ×6.2 ± 0.4 µm, n=10). This description fits that of the original description of *P. latipes* (see legend of figure 8). Scale bars: B-C = 200 µm, D = 100 µm, E-F, L = 20 µm, G-K, M = 10 µm.
